# RNA-seq reveals the RNA binding proteins, Hfq and RsmA, play various roles in virulence, antibiotic production and genomic flux in *Serratia* sp. ATCC 39006

**DOI:** 10.1186/1471-2164-14-822

**Published:** 2013-11-22

**Authors:** Nabil M Wilf, Adam J Reid, Joshua P Ramsay, Neil R Williamson, Nicholas J Croucher, Laurent Gatto, Svenja S Hester, David Goulding, Lars Barquist, Kathryn S Lilley, Robert A Kingsley, Gordon Dougan, George PC Salmond

**Affiliations:** Department of Biochemistry, University of Cambridge, Tennis Court Road, Cambridge, CB2 1QW UK; The Wellcome Trust Sanger Institute, Wellcome Trust Genome Campus Hinxton, Cambridge, UK; Cambridge Centre for Proteomics, University of Cambridge, Cambridge, UK; School of Biomedical Sciences, CHIRI Biosciences Research Precinct, Curtin University, Perth, WA Australia

## Abstract

**Background:**

*Serratia* sp. ATCC 39006 (S39006) is a Gram-negative enterobacterium that is virulent in plant and animal models. It produces a red-pigmented trypyrrole secondary metabolite, prodigiosin (Pig), and a carbapenem antibiotic (Car), as well as the exoenzymes, pectate lyase and cellulase. Secondary metabolite production in this strain is controlled by a complex regulatory network involving quorum sensing (QS). Hfq and RsmA (two RNA binding proteins and major post-transcriptional regulators of gene expression) play opposing roles in the regulation of several key phenotypes within S39006. Prodigiosin and carbapenem production was abolished, and virulence attenuated, in an S39006 ∆*hfq* mutant, while the converse was observed in an S39006 *rsmA* transposon insertion mutant.

**Results:**

In order to define the complete regulon of Hfq and RsmA, deep sequencing of cDNA libraries (RNA-seq) was used to analyse the whole transcriptome of S39006 ∆*hfq* and *rsmA*::Tn mutants. Moreover, we investigated global changes in the proteome using an LC-MS/MS approach. Analysis of differential gene expression showed that Hfq and RsmA directly or indirectly regulate (at the level of RNA) 4% and 19% of the genome, respectively, with some correlation between RNA and protein expression. Pathways affected include those involved in antibiotic regulation, virulence, flagella synthesis, and surfactant production. Although Hfq and RsmA are reported to activate flagellum production in *E. coli* and an adherent-invasive *E. coli hfq* mutant was shown to have no flagella by electron microscopy, we found that flagellar production was increased in the S39006 *rsmA* and *hfq* mutants. Additionally, deletion of *rsmA* resulted in greater genomic flux with increased activity of two mobile genetic elements. This was confirmed by qPCR and analysis of *rsmA* culture supernatant revealed the presence of prophage DNA and phage particles. Finally, expression of a hypothetical protein containing DUF364 increased prodigiosin production and was controlled by a putative 5′ *cis*-acting regulatory RNA element.

**Conclusion:**

Using a combination of transcriptomics and proteomics this study provides a systems-level understanding of Hfq and RsmA regulation and identifies similarities and differences in the regulons of two major regulators. Additionally our study indicates that RsmA regulates both core and variable genome regions and contributes to genome stability.

**Electronic supplementary material:**

The online version of this article (doi:10.1186/1471-2164-14-822) contains supplementary material, which is available to authorized users.

## Background

*Serratia* spp. are Gram-negative enterobacteria and are opportunistic human, insect, and plant pathogens. *Serratia* sp. ATCC 39006 (S39006) produces the secondary metabolite, prodigiosin (Pig) which is a linear tripyrrole that confers a characteristic red pigment in colonies of this strain. Prodigiosin is a member of the prodiginines which are of potential clinical and translational interest for their antimicrobial and immunosuppressant properties. Furthermore, an analogue is currently in phase I/II clinical trials for cancer chemotherapy [1]. Additionally, S39006 produces a β-lactam antibiotic; a carbapenem (1-carbapen-2-em-3-carboxylic acid; Car) [2], and the plant cell wall-degrading exoenzymes, pectate lyase and cellulase [3]. S39006 demonstrates a broad host-range capacity for pathogenesis, being virulent in both animal (*Caenorhabditis elegans*) and plant (potato) host infection models [4, 5].

The enzymes for prodigiosin and carbapenem production are encoded, respectively, by the *pigA-O* and *carA-H* biosynthetic operons [3, 6]. Previous studies have characterized the pathways involved in the biosynthesis and regulation of prodigiosin, revealing a complex regulatory network involving at least 20 genes - some of which also co-regulate carbapenem production [7, 8]. Multiple environmental inputs, including quorum sensing (QS) [3] and phosphate and gluconate levels [9], regulate the production of prodigiosin. QS is a mechanism by which bacteria regulate gene expression in response to population cell density via detection of a diffusible signaling molecule. S39006 possesses a LuxIR-type QS system encoded by the *smaIR* locus. In S39006, the major signaling molecule synthesized by SmaI is *N*-butanoyl-L-homoserine lactone (BHL) [6]. SmaR is one of several atypical LuxR-type regulators the activity of which is inhibited by their cognate acyl homoserine lactones (AHLs) [10]. At high cell density, SmaR-mediated repression of the *pig* and *car* gene clusters is de-repressed by rising BHL levels, resulting in the biosynthesis of prodigiosin and the carbapenem. CarR is a dedicated LuxR homologue that controls, in a uniquely AHL-independent manner, the transcriptional activation of the *car* operon [6, 11].

Recently, the RNA-binding proteins and post-transcriptional regulators, RsmA and Hfq, were shown to play opposing roles in the regulation of antibiotic production and pathogenesis in S39006 [12] (N. Williamson, submitted). An S39006 ∆*hfq* mutant failed to make the prodigiosin or the carbapenem and virulence was attenuated in both potato and *C. elegans* infection models [12], while an S39006 *rsmA* transposon insertion mutant showed elevated prodigiosin production and enhanced virulence in these models (N. Williamson, submitted).

In *Pseudomonas aeruginosa* and *P. fluorescens*, RsmA, the homologue of *E. coli* CsrA, plays an important role in regulation of virulence and biocontrol factor production [13]. RsmA represses expression of specific genes by binding to the 5′ untranslated region (UTR) of target mRNAs, preventing translation. This repression is relieved by RsmA binding to the small, noncoding regulatory RNA (sRNA) RsmB, instead of its target mRNAs [14]. Hfq, a hexameric protein that forms a doughnut-like structure, is an RNA chaperone that facilitates the complementary base-pairing between *trans*-acting sRNAs and target mRNAs. Increasing evidence shows that Hfq and its dependent sRNAs play a fundamental role in the regulation of stress response and pathogenesis [15].

To define and investigate the regulon(s) of Hfq and RsmA, deep sequencing of cDNA libraries (RNA-seq) was used to analyse changes in gene expression across the whole transcriptome of S39006 ∆*hfq* and *rsmA*::Tn, and were compared to global changes in the proteome examined by an LC-MS/MS approach of iTRAQ-labelled peptides. Gene expression analysis revealed that Hfq and RsmA directly or indirectly regulate 4% and 19%, respectively, of the genome at the level of RNA - with some correlation between RNA and protein expression. Several pathways were affected including antibiotic regulation, virulence, and flagella synthesis. In particular, it was observed that RsmA is key to the stability of two mobile genetic elements. The combination of transcriptomics and proteomics provides a systems-level understanding of Hfq and RsmA regulation in S39006.

## Results and discussion

### Differential expression analysis of the transcriptome of the S39006 hfq and rsmA mutants

**Figure 1:**
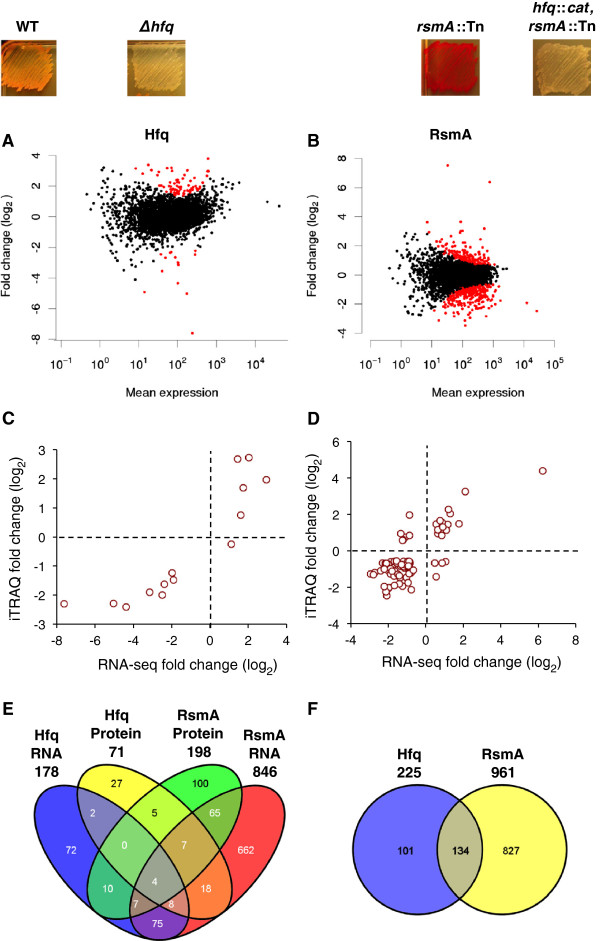
**Analysis of differential gene expression in the** ***hfq*** **and** ***rsmA*** **mutants.** Hfq is an activator while RsmA is a repressor of prodigiosin production as indicated by the level of pigment of the mutant strains. Generation of the *hfq::cat*, *rsmA*::Tn double mutant resulted in repression of pigment production indicating that the *hfq* mutant is epistatic over the *rsmA* mutant. **A** **-B**. The R package, DESeq, was used to analyze differential gene expression of the RNA-seq data. A scatter plot of the fold change (log_2_ ratio) versus mean expression is shown for the *hfq* **(A)** and *rsmA* **(B)** mutants. The red circles indicate genes identified as differentially expressed with a 1% false discovery rate according to the method of the Benjamini-Hochberg multiple testing adjustment. **C-** **D**. The RNA-seq and iTRAQ-proteomic datasets for the *hfq* **(C)** and *rsmA* **(B)** mutants were compared by plotting the fold change (log_2_ ratio) of differentially expressed genes. 13 and 73 genes had fold changes in the same direction in the *hfq* and *rsmA* datasets, respectively, which gave a Pearson correlation coefficient > 0.90 and >0.85. **E-** **F**. The Venn diagrams illustrate the overlap between the RNA-seq and protein iTRAQ datasets for the *hfq* and *rsmA* mutants to identify co-regulated genes **(E)**. The uniquely regulated genes in the RNA and protein datasets for each mutant was combined to produce a list of all the genes that were significantly differentially expressed. The combined datasets for each mutant was also compared to identify the overlap between them **(F)**.

To compare the transcriptomes of the S39006 WT and the *Δhfq* (NMW8) and *rsmA*::Tn (NMW7) mutants, total RNA was first isolated from early stationary phase cultures of three biological replicates, when antibiotic production is maximal. Single-stranded cDNA libraries were assembled and sequenced (Table 1). Differential expression analysis using DESeq between the WT and the mutants of *hfq* and *rsmA* showed that, with a false discovery rate (FDR) correction of 1%, 86 genes (14 decreased; 72 increased) or 2% of the transcriptome were identified as significantly different in the *hfq* mutant, while expression of 504 genes (339 decreased; 65 increased) or 11% of the transcriptome was identified in the *rsmA* mutant (Figure 1A-B). The number of genes identified in the *hfq* mutant was less than the 785 genes (or 18% of the genome) altered in an *hfq* mutant of the taxonomically related enterobacterium, *Salmonella* Typhimurium [16]. This may be attributable to the increased variance of the replicates used in the analysis as illustrated by the atypical scatter plot of gene expression for the *hfq* mutant compared to the *rsmA* mutant (Figure 1A-B).

**Table 1:** **RNA-sequencing data mapped to the S39006 genome**

Flowcell ID_lane	4577_1	4577_2	4583_1	4577_3	4577_5	4583_2	4577_6	4577_7	4583_3
Library	WT-1	WT-2	WT-3	hfq-1	hfq-2	hfq-3	rsmA-1	rsmA-2	rsmA-3
Total reads (× 10^6^)	61.8	48.1	41.2	53.6	34.1	44.3	47.5	43.2	17.5
Reads mapped (× 10^6^)	27.9	26.5	26.9	31.7	20.1	28.1	4.2	7.1	8.5
% Reads mapped	45%	55%	6%	59%	59%	64%	9%	16%	49%

**Figure 2:**
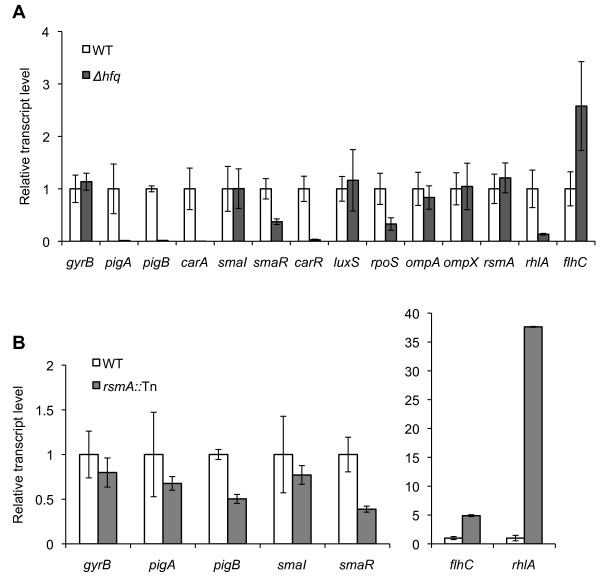
**qRT-PCR showing differential gene expression.** Gene expression for *Δhfq* **(A)** and *rsmA*::Tn **(B)** is measured as transcript levels relative to WT at early stationary phase growth. *gyrB* is included as a negative control. Values represent average gene expression ± SD from three independent experiments except values for genes *smaI* and *flhC* which were determined from two independent experiments.

Differentially expressed genes identified by DESeq were correlated with relative transcript levels determined by qRT-PCR confirming that the analysis by DESeq provides accurate quantitative estimates of fold-change in the mutant versus the WT. The comparison also guided the re-adjustment of the FDR cut-off values in order to evaluate a wider number of differentially expressed genes. The same RNA samples used for RNA-seq were analysed by qRT-PCR to measure the relative expression of genes, some of which were previously reported to be differentially regulated in the *hfq* and *rsmA* mutants (Figure 2; Additional file 1: Table S3). For the *hfq* mutant, these included the *pigAB* genes involved in prodigiosin production and the QS regulators, *smaR* and *carR*[12]. For the *rsmA* mutant, these included the *rhlA* and *flhC* genes essential for swarming and swimming motility, respectively [17]. Of the eight genes determined as differentially expressed in the *hfq* mutant by qRT-PCR, six were corroborated by the RNA-seq analysis, five with an FDR of <5% and one with an FDR of <10%. Of the four genes determined as differentially expressed in the *rsmA* mutant by qRT-PCR, three were corroborated by the RNA-seq analysis with an FDR of < 0.001%. As a result of the comparison of the differential analysis by qRT-PCR and RNA-seq, and because of the increased variation in the *hfq* RNA-seq data, an FDR threshold of 10% and 5% was used (for the *hfq* and *rsmA* mutants, respectively) as a cut-off for identifying and examining differentially expressed genes. At an FDR threshold of 10%, 178 genes (30 decreased, 148 increased) were differentially expressed in the *hfq* mutant, or 4.0% of the predicted 4418 ORFs in the genome. At an FDR threshold of 5%, 846 genes (523 decreased, 323 increased) were differentially expressed in the *rsmA* mutant (representing 19.1% of the genome).

### Proteome-transcriptome correlations and changes in the car and pig clusters

The results of the RNA-seq differential analysis were compared with changes in global protein expression using quantitative proteomics. Protein was extracted from the WT and *hfq* strains grown under the same conditions as those used in the RNA-seq analysis and changes in protein expression were investigated using an LC-MS/MS approach of iTRAQ-labelled peptides. Peptides mapped to 1,369 proteins in total and 486 proteins met a cut-off of two or more mapped unique peptides of which 36 (23 decreased; 13 increased) were differentially expressed (*P* value < 0.1) [18]. The higher cut-off of *P* value < 0.1 was used due to the increased variance. 14 of the 36 proteins were also differentially expressed in the *hfq* RNA-seq dataset (FDR < 0.1) (Figure 1C; Additional file 2: Table S5). A comparison of the fold changes (log_2_ ratio) showed the direction of change was the same for 13 genes (8 decreased and 5 increased at the level of both RNA and protein) which gave a Pearson correlation coefficient of >0.90. Expression of one gene was decreased at the level of protein production but increased for the mRNA transcript, which may indicate a post-transcriptional effect on regulation.

The *rsmA* mutant proteome was also investigated using the same iTRAQ-based approach in a different study (N. Williamson and J. Ramsay, unpublished). Samples used in the analysis were grown to mid stationary phase (T = 12 hr) compared to early stationary phase for samples investigated by RNA-seq (T = 10 hr). Although the time points are different at which the transcriptome and proteome were investigated during stationary phase, it was still noteworthy to determine if there was any overlap between the two datasets in terms of the direction of fold change at the level of RNA and protein for individual genes. A comparison of the differentially expressed genes in the RNA-seq (FDR < 0.5) and iTRAQ datasets (FDR < 0.1) identified 83 genes that were differentially expressed at the level of both RNA and protein (Figure 1D). The fold changes of 73 of these genes were in the same direction for both RNA and protein (59 decreased; 14 increased), and had a Pearson correlation coefficient of >0.85. For the 10 genes that were regulated in different directions at the level of RNA and protein, 4 showed increased transcript levels but decreased protein expression, and 6 showed decreased transcript levels but increased protein expression. The *pigA* and *pigB* genes were among the latter 6 genes and showed reduced (0.51 and 0.43-fold) RNA and increased (1.71 and 1.96-fold) protein production. qPCR analysis confirmed that *pigB* showed decreased mRNA production. This suggests that altered post-transcriptional regulation of the *pig* operon might explain that, despite decreased RNA levels, increased translation of prodigiosin biosynthetic enzymes leads to greater prodigiosin production and the observed hyperpigmented phenotype of the *rsmA* mutant.

Differentially expressed genes in the RNA-seq and protein iTRAQ datasets for the *hfq* and *rsmA* mutants were compared to identify potentially co-regulated genes (Figure 1E). The biosynthetic genes *pigA* and *carABC* were the four genes altered at both an RNA and protein level in both mutants. The combined RNA and protein datasets for each mutant were compared and this revealed that 134 genes were co-regulated by Hfq and RsmA (Figure 1F). Several genes within various genetic clusters across the genome were either up- or down-regulated in the two mutants (Table 2).

**Table 2:** **A selection of differentially expressed genetic clusters in the** ***hfq*** **and** ***rsmA*** **mutants**

Genetic clusters	***hfq***		***rsmA***	
	RNA^1^	Protein^1^	RNA^1^	Protein^1^
Prodigiosin production	↓	↓	↓	↑
Carbapenem production	↓	↓	↓	↑
Type II secretion	−	−	↑	−
Type IV pilus	−	−	↑	−
Electron transport	↑	−	−	−
Flagellar production	↑	−	↑	↑
Prophage elements	−	−	↑	↑

^1^Differentially expressed genes were identified in the transcriptomic and proteomic datasets. Up and down arrows indicate increases and decreases in expression at the level of RNA or protein. Fold changes for individual genes in these clusters as well as genes in other pathways are listed in Additional file 1: Table S4. A complete list of all genes identified as differentially expressed are listed in Additional file 2: Table S5.

Expression of the *pig* and *car* biosynthetic clusters was examined further in the *hfq* and *rsmA* mutants (Table 3). For the *rsmA* mutant, expression of other genes in the *pig* cluster (*pigCDH*) was decreased 0.57 to 0.68-fold at the level of RNA. However, in contrast, *pigF* expression was increased at the protein level providing further evidence that the increased prodigiosin production of an *rsmA* mutant may be due to altered post-transcriptional regulation. Another possibility is that substrates of the respective *pig* enzymes may also be more readily available. However, expression of genes in the *car* cluster was consistently decreased at both an RNA (*carABCDF*; fold change 0.21-0.40) and protein (*carABC*; fold change 0.46-0.51) level in the *rsmA* mutant. Unfortunately, due to interference from surfactant production, it was not possible to measure production of the carbapenem accurately in the *rsmA* mutant by simply observing the zone of inhibition of an *E. coli* sensor strain in a plate bioassay. For the *hfq* mutant (which fails to make the carbapenem antibiotic) the results were straightforward and showed that expression of the *pig* and *car* genes was significantly decreased at both an RNA and protein level. In summary, there is significant consistency for differential expression at the transcriptomic and proteomic levels in the *hfq* and *rsmA* mutants, though with a greater degree of overlap observed for the *rsmA* mutant.

**Table 3:** **Differentially expressed genes in the** ***pig*** **and** ***car*** **clusters**

Gene	***hfq*** RNA^1^	***hfq*** Protein^1^	***rsmA*** RNA^1^	***rsmA*** Protein^1^	Description
Pig cluster					
or1369	0.11	0.24	0.51	1.71	PigA;
or1370	0.21	-	0.43	1.96	PigB;
or1371	0.26	0.37	0.57	-	PigC;
or1372	0.27	0.32	0.68	-	PigD;
or1373	-	0.32	-	-	PigE;
or1374	-	0.41	-	1.55	PigF;
or1376	-	-	0.67	-	PigH;
Car cluster					
or3484	0.06	-	-	-	CarR;
or3483	0.03	0.18	0.30	0.51	CarA;
or3482	0.05	0.17	0.31	0.46	CarB;
or3481	0.01	0.18	0.21	0.47	CarC;
or3480	0.16	-	0.40	-	CarD;
or3478	0.13	-	0.22	-	CarF;

^1^Fold changes were calculated as the ratio of mutant to WT. Differentially expressed genes were identified with an FDR threshold of 10% for RNA levels of *hfq* and protein levels of *rsmA*. A 5% FDR threshold was used for RNA levels of *rsmA. hfq* protein levels were identified with a *P* value < 10%.

### Flagella production increased

**Figure 3:**
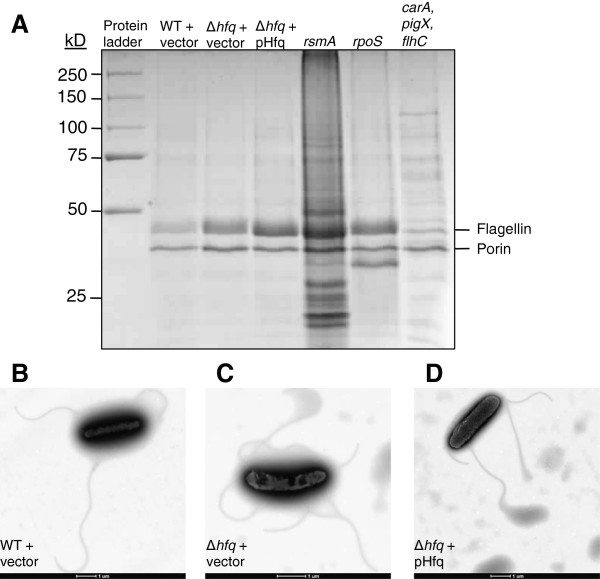
**Analysis of flagellin. A**. Extracts of flagella were analyzed by SDS-PAGE. Two abundant protein bands were identified by mass spectrometry (MALDI-TOF) as flagellin (or3159, 41.4 kD) and a porin (or3073, 38.8 kD). **B-** **D**. Transmission electron micrographs of WT + vector **(B)**, Δ*hfq* + vector **(C)**, and Δ*hfq* + pHfq **(D)** are shown with the scale bar at the bottom of each image indicating a length of 1 μm. Samples were grown to early stationary phase the same as those used in the RNA-seq. The vector used was pQE80oriT and pHfq refers to pNB36. The strains analyzed were NMW9 (Δ*hfq*), NMW7 (*rsmA*), NMW25 (*rpoS*), and NWX21 (*carA*, *pigX*, *flhC*).

Additional genetic pathways affected in the *hfq* mutant include the hierarchical genetic system for production of flagella. Analysis of the RNA-seq data showed that expression of 22 of 35 genes involved in flagella production (which are clustered in the genome and are contained in various operons on either strand) was increased from 2.5 to 9.1-fold, despite a decrease in swimming and swarming motility. In order to explore this result, an extract of flagellar proteins was examined by SDS-PAGE analysis and major protein bands were identified by mass spectrometry (Figure 3A). *flhC* is located in the flagellar master regulator operon, and the control strain with a mutation in *flhC* gave a reduced band of flagellin protein and, in a previous study, was shown by electron microscopy to possess no flagella [17]. The results suggest that flagellin levels were increased significantly in the Δ*hfq* mutant and in the *rsmA* and *rpoS* mutants which displayed increased motility [19]. The RNA-seq analysis of the *rsmA* mutant also revealed that expression of 19 of the 35 flagellar genes was increased from 1.6 to 4.0-fold along with the flagellar sigma factor, *rpoF*. Furthermore, iTRAQ analysis showed that flagellin protein was increased (Additional file 1: Table S4). The second gel band was identified as a porin protein, which was increased in the *hfq* mutant. Although, complementation of the *hfq* mutant restored swimming motility, expression of Hfq *in trans* did not reduce flagellin and porin to WT levels. Electron microscopy of the WT, *hfq* mutant, and complemented strain showed no significant differences in the presence of flagella on the cell surface (Figure 3B-D). This is in contrast to the role Hfq plays in flagellum production in *S. typhimurium* where 87% of the flagellar genes were downregulated in an *hfq* mutant which was impaired for swimming motility, and Hfq was shown to bind to *flhDC* mRNA [16]. Also, no flagella were present in an electron micrograph of an *E. coli hfq* mutant [20]. However, the species-specific role of Hfq in the regulation of motility is illustrated by the increased swarming motility of a *Y. pseudotuberculosis hfq* mutant as well as increased biosurfactant production [21]. Therefore, it will be interesting to explore further the role of Hfq in the regulation of flagellum production in S39006, and the cause of the decreased motility despite an increase in flagellar gene expression.

### Regulation of the electron transport genetic operon

Another set of genes with increased transcript levels in the *hfq* mutant include seven of 13 genes (2.2 to 3.5-fold) in an operon encoding an NADH dehydrogenase, whereas five of the genes were decreased 0.31 to 0.62-fold in the *rsmA* mutant. This operon is homologous to the *nuo* operon of *E. coli*. This encodes the membrane protein NADH:ubiquinone oxidoreductase I (NDH-1) which is one of two NADH deyhydrogenases, the second being (NDH-2). NDH-1 and NDH-2 catalyze the transfer of an electron from NADH to ubiquinone as part of the electron transport chain in aerobic respiration. However, only electron flow catalysed by NDH-1 generates an electrochemical gradient, while NDH-2 is the predominant respiratory source of H_2_O_2_ and O^–^_2_, although H_2_O_2_ is primarily formed from a source outside the respiratory chain in *E. coli*[22, 23]. Similarly, a related protein, the NADPH-dependent quinone oxidoreductase, *qor*, was found to be decreased at the level of both RNA and protein production in the *S. typhimurium hfq* mutant [16, 24], and *E. coli* Hfq was shown to be associated with *qor* DNA [25]. These results suggest that Hfq could mediate the regulation of aerobic respiration and the transfer of electrons to the quinone pool within the cell.

### Regulation of prodigiosin regulators

Immediately upstream of the *nuo* operon is a LysR-family transcriptional regulator referred to as PigU in S39006, expression of which was increased at the level of RNA (3.4-fold) and protein (2.85-fold) in the *hfq* mutant. This regulator is a homologue of HexS and HexA (**h**yperproduction of **ex**oenzymes; 74% and 82% identical) from *S. marcescens* 274 and *P. atrosepticum* SCRI1043 (*Pat*), respectively. HexS was shown to bind to the *pigA* promoter and reduce prodigiosin production [26], and expression of HexA from *Pat* and the related *P. carotovorum* (*Pcc*) in S39006 repressed prodigiosin and carbapenem production [27]. Therefore, the increased levels of PigU in the *hfq* mutant most likely contribute to the repression of prodigiosin production. *Pcc* HexA also represses exoenzyme production and binds to the promoter of *pelC* encoding a pectate lyase [27] and it represses the production of *rpoS* and RsmB, the small RNA antagonist of RsmA [28]. The HexA homologue in *E. coli* (LrhA) was shown to repress *rpoS* expression at the level of translation and represses the transcription of the sRNA RprA [29]. Therefore, increased PigU in the *hfq* mutant may contribute to the decrease in *rpoS* transcript levels and exoenzyme production.

Expression of several additional regulators of pigment production, identified in previous studies, were significantly decreased in the *rsmA* mutant. These include *rap*, *pstS*, *phoU*, *rpoS*, and *vfmE*. Rap is a transcriptional regulator of the SlyA family and an activator of secondary metabolism [30]. *pstS* and *phoU* form part of the *pstSCAB*-*phoU* operon which, when deleted, causes increased hyperpigmentation [3]. *rpoS* is a sigma factor which is a repressor of secondary metabolism [19, 31]. Finally, *vfmE* encodes an AraC-type regulator which is an activator of secondary metabolism [32].

### Secretion and pili system regulated by rsmA

Secretion and pili systems involved in exporting virulence factors and attachment, respectively, were also identified as differentially expressed in the *rsmA* mutant. S39006 encodes Type I, II and III secretion systems (T1SS, T2SS and T3SS). The *rsmA* mutant showed elevated expression of genes involved in T2SS (1.8 to 3.2-fold) and Type IV pilus (T4P) genes (1.4 to 1.5-fold), whereas a cluster of T3SS genes was unaffected. Four genes in a cluster encoding a Type II (Out) secretion system showed increased expression in the *rsmA* mutant. However, SecY production was reduced and this protein is part of the SecYEG translocon that acts as a conduit for proteins moving across the inner membrane into the periplasm for subsequent secretion across the outer membrane by the T2SS machinery.

Further investigation of the T4P genes revealed that S39006 possesses an 11 gene cluster (*pilLMNOPQRSUVW*; 10,806 bp) of which 5 show increased expression in the *rsmA* mutant. The *pil* cluster resides within an 82.1 kb putative pathogenicity island (PAI) spanning or 1466 to or 1551 (86 genes total) and is most closely related to the *pil* cluster within the *Yersinia* adhesion pathogenicity island (YAPI; 98 kb) found in *Y. pseudotuberculosis* and *Y. enterocolitica*, but not *Y. pestis*[33]. The percent identity with the 11 Pil proteins from *Y. pseudotuberculosis* ranged from 43% to 61%. The *pil* gene cluster is also found within the *Salmonella enterica* pathogenicity island SPI-7 (134 kb), the *Pat* horizontally acquired island HAI-2 (97.6 kb) [34, 35], and the recently sequenced *Erwinia* strain Ejp617 which causes bacterial shoot blight of pear in Japan [36]. However, the S39006 *pil* cluster is not present in related *Serratia* species. The S39006 PAI contains the characteristic features of a PAI which include: i) insertion between a tRNA-Phe gene downstream of or1551 and a 50 bp partially duplicated copy before or1466 (similar to YAPI and SPI-7), ii) a G + C content of 52.9% which differs from the rest of the genome (49.2%), and, iii) a number of genes encoding products associated with mobile genetic elements, such as integrases and transposases. The S39006 PAI carries 86 ORFs which include a type-I restriction modification system and the capacity to express CRISPR-associated proteins.

**Figure 4:**
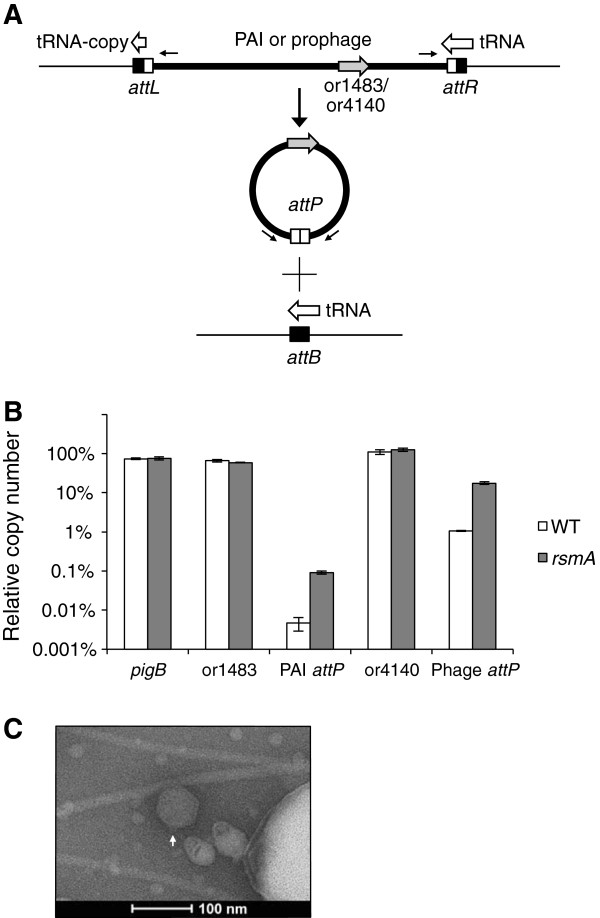
**Analysis of phage and PAI excision and TEM of phage. A**. Excision and circularization of the PAI and prophage elements were confirmed by amplification of the *attP* site by PCR using primers (black arrows) that faced out of the 5′ and 3′ ends of the genomic islands (data not shown). The PAI and prophage are both inserted between a tRNA gene at the 3′ end and partially duplicated copy at the 5′ end. **B**. qPCR analysis of genomic DNA samples showed that the frequency of excision of the PAI and prophage is higher in the *rsmA* mutant compared to WT. Copy number of *pigB* was included as a negative control. or1483 and or4140 are cargo genes on the PAI and prophage, respectively. Copy number was determined as the percentage of cells containing the gene or *attP* site relative to the copy number of the internal reference gene *gyrB*. Values represent average gene expression ± SD from three independent experiments. **C**. TEM image of a putative podoviridae-like phage particle (65–70 nm in diameter) isolated from the culture supernatant of the *rsmA* mutant. The arrow indicates what may be a very short tail at the apex.

Excision of the PAI from the genome and the formation of an extrachromosomal circular intermediate were confirmed by PCR analysis. Primers were designed facing outwards from the 5′ and 3′ ends of the PAI integration sites, *attL* and *attR*, respectively (Figure 4A). A single PCR product was obtained indicating that the PAI excised from the host genome and formed a circular intermediate, allowing DNA amplification across the *attP* site. qPCR analysis was used to determine the copy number of one of the genes of the *pil* cluster, or1483, as well as that of the *attP* site (Figure 4B). The results showed that there was no increase in the copy number of or1483 but a 23-fold increase for the *attP* site. This suggests that there is increased excision and circularization but not replication of the PAI.

Some evidence suggests that the *pil* cluster promote pathogenesis. The *S. enterica pil* cluster was shown to encode type IVB pili and a *pil*^*–*^ mutant was significantly reduced for invasion into human intestinal cells [37]. Additionally, a *Y. pseudotuberculosis pil*^*–*^ mutant was delayed in the killing of mice when administered orally, however it was as virulent as the parental strain when administered intravenously. A survey for the presence of the *pil* locus showed that it was present in only 38 of 92 (41%) tested strains of *Y. pseudotuberculosis*. Collyn *et al.*[38] suggested that the *pil* cluster may form an “adaptation pathogenicity island” allowing the bacteria to colonize a wider variety of hosts.

### Regulation of virulence factors and secreted products

Secreted virulence factors were identified as differentially expressed in the *hfq* and *rsmA* mutants. In the *rsmA* mutant the expression of three genes involved in pectate lyase production was decreased whereas the mRNA of pectate lyase L was increased 4.67-fold which is consistent with the 8.44-fold increase in secreted Pel activity relative to the WT (N. Williamson, in preparation). However, cellulase expression was reduced although secreted Cel activity was marginally increased 1.31-fold. Cellulase and pectate lyase are secreted by the T2SS and the increased expression of the Type 2 (Out) system may explain the apparent increase in detectable Cel activity, despite a decrease in expression of the cognate gene. Interestingly, the gene with the highest fold change (184-fold) is an uncharacterized protein (or1354) that shares 44% aa similarity to the δ-thermostable haemolysin (δ-VPH) of *V. cholerae* and *parahaemolyticus* species. The *Vibrio* species contain four families of haemolysins, including TDH of *V. parahaemolyticus* and HlyA of *V. cholerae* which are closely associated with virulence through lysis of cells, notably erythrocytes [39]. However, the role of some haemolysins, such as δ-VPH, is not clear. A study examining the role of δ-VPH in *V. cholerae* O1 showed that it is a 22.8 kD protein with haemolytic activity on sheep erythrocytes [40]. Haemolytic activity was observed in both whole-cell lysates and supernatant fractions despite the absence of an identifiable signal peptide. Similarly, or1354 is predicted to encode a 22.6 kD protein, but no signal peptide was identified.

Additionally, or1354 is part of a putative operon of four genes (or1352-5) all of which were among the top ten genes with the highest fold-change in the RNA-seq dataset of the *rsmA* mutant. Proteins encoded by the four genes are most similar to proteins from the plant endosymbiont, *Pseudomonas putida* W619, and various soil species isolates of *Burkholderia*. The genes forming the operon (in addition to or1354) include: or1352 encoding taurine catabolism dioxygenase (TauD/TfdA) which in *E. coli* allows the utilization of taurine as a sulphur source under conditions of sulphate starvation; or1353 encoding an AMP-dependent synthetase and ligase; and or1355 encoding a drug resistance transporter of the Bcr/CflA subfamily.

Additional categories of genes that showed altered expression in the *rsmA* mutant include efflux transporters important in drug resistance, outer membrane proteins, sigma factors, and stress response genes. The gene or1713 had the second highest increase in expression (83-fold) in the RNA-seq dataset and was matched by a 21-fold increase in protein. It encodes a small putative exported protein (125 aa) and a conserved domain search indicates that it has a helix-hairpin-helix repeat region. Members of this domain subfamily include competence protein ComEA (for transformation by exogenous DNA).

Expression of or0696 (encoding gas vesicle protein GvpA1) was decreased by a factor of 10 in the *rsmA* mutant, which matched the significant decrease in expression of a *gvpA1:uidA* reporter in an *rsmA*::Tn mutant background [41]. Expression of or1347 encoding QueF (involved in queuosine biosynthesis) was reduced by about half in both the *rsmA* and *hfq* mutants. These results suggest RsmA regulates very diverse phenotypes including genes connected with the production of intracellular gas vesicles and queuosine products.

Genes involved in surfactant production were also affected. It has been shown that *rhlA*, encoding a homologue of *Pseudomonas aeruginosa* RhlA involved in rhamnolipid biosynthesis, showed decreased expression in an *hfq* mutant and increased expression in a *rsmA* mutant [12, 17]. Likewise, it was shown in the *rsmA* dataset that *rhlA* expression was increased at the level of RNA and protein. Additional genes predicted to be involved in surfactant production were also investigated. The surfactant, serrawettin W2, produced by *S. marcescens* Db10 (Sma) is encoded by the *swrA* gene (> 17 kb), predicted to encode a nonribosomal peptide synthase (NRPS), and was identified as the factor inducing the worm, *C. elegans*, to avoid bacterial lawns of Sma [42]. Interestingly, *C. elegans* did not display lawn avoidance behaviour toward the S39006 *hfq* mutant compared to WT, suggesting that surfactant production is affected by the *hfq* deletion [12]. A BLAST search for homologues of *swrA* identified or2773 and an operon of five genes (or3032-28; 19.8 kb). The first gene (or3032) in the operon is predicted to encode a cyclic peptide transporter with six transmembrane helices suggesting that it is an inner membrane protein that exports the product synthesized by or3032-28. Expression at the level of RNA was elevated for or3031, or3030, and or2773 (1.64 to 2.30-fold) in the *rsmA* mutant. qRT-PCR analysis confirmed that expression of or3031 and or2773 was moderately increased in the *rsmA* mutant, with no significant change for or3030, whereas, for the *hfq* mutant, or3231-30 showed decreased expression (0.5-fold) with no change for or2773 (Additional file 1: Figure S1). Interestingly, expression of or3032 (encoding the putative transporter) was elevated in the *hfq* mutant but decreased in the *rsmA* mutant. Therefore, a decrease in production of the NRPS genes was associated with an increase in expression of the transporter, and *vice versa*.

### RsmA regulates prophage expression

Perhaps one of the most surprising observations for the *rsmA* mutant is the increased expression of three putative prophage clusters encoding bacteriophage or related genes, including the CI repressor, CII regulator, reverse transcriptase, and lysozyme. These results suggest that RsmA regulates lysogeny in this strain and that the absence of *rsmA* results in increased expression of prophage genes and perhaps a physiological switch into the lytic cycle.

Some prophages are known to insert into tRNA sites. Of the three prophage clusters in this strain, the third prophage element spans 40.7 kb from or4140 to or4196 (57 genes total) and or4196 is a putative phage integrase. 39 of the 57 genes in this island showed significantly increased expression (1.52 to 3.90-fold) at the level of RNA in the *rsmA* mutant. This was also confirmed by qRT-PCR for or4140 which showed elevated expression (6.89-fold) in the *rsmA* mutant, but with no change for the *hfq* mutant (Additional file 1: Figure S1). A tRNA-Thr gene is located immediately downstream of or4196 and a BLAST search of the tRNA sequence revealed a 46 bp partially duplicated copy before or4140. This suggested that tRNA-Thr is the integration site for this prophage. Prophages that excise from the genome circularize and reconstitute the phage attachment site (*attP*) while the bacterial genome recombines and forms the bacterial attachment site (*attB*). Amplification by PCR of the *attP* and *attB* sites from WT genomic DNA confirmed the excision and circularization of the prophage through recombination in the genome (Data not shown; Figure 4A). qPCR analysis of the relative copy number of the *attP* site in the *rsmA* mutant versus the WT samples revealed that there was a 17-fold increase in the frequency of excision in the rsmA mutant; although there was no increase in copy number for or4140 (a cargo gene), suggesting that the prophage does not replicate within the cell (Figure 4B).

To determine if phage particles were released from the cell, cultures of S39006 were grown to early stationary phase and a phage DNA extraction kit was used to analyse the contents of filtered supernatants. Prophage DNA (but neither host DNA nor DNA of the PAI) was amplified by PCR analysis of the extracted DNA. Additionally, transmission electron microscopy (TEM) of the culture supernatant showed small podoviridae-like phage particles in the *rsmA* (but not WT) sample - with hexagonal heads 65-70 nm in diameter with some displaying a very short tail at one apex (Figure 4C). Nevertheless, none of the bacterial strains tested showed susceptibility to infection by this or other induced prophages released by S39006. The strains tested included S39006, *S. marcescens* 274, *P. atrosepticum* SCRI1043, *P. carotovorum* subsp. *carotovorum*, *Citrobacter rodentium* DBS100, and *P. aeruginosa* PA01. In summary, the evidence suggests that deletion of *rsmA* results in increased excision of the prophage, expression of the cargo genes, and release of the prophage from the cell.

### Identification of a cis-acting regulatory RNA element

**Figure 5:**
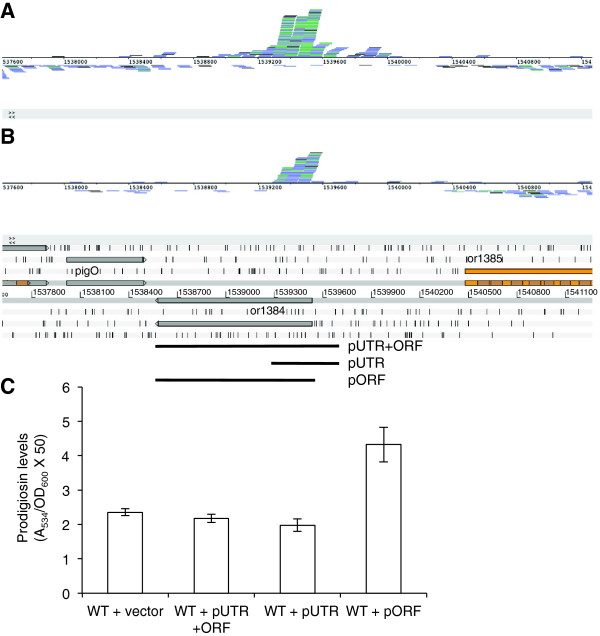
**DUF364 expression increases prodigiosin production and is controlled by a putative 5′** ***cis*** **-acting regulatory RNA element. A-** **B**. RNA-seq reads are shown mapped to the conserved hypothetical ORF (or1384; DUF364) which is divergent to *pigO* of the *pigA-O* cluster. The cumulative coverage is shown for the three replicates of the WT **(A)** and the *hfq* mutant **(B)**. A peak was also observed in the replicates of *rsmA* (data not shown). The coverage is shown as raw reads aligned to the reference genome sequence in BAM strand stack view in the genome browser Artemis with the annotation displayed underneath. The colour scheme for the reads is blue for paired reads; black for single reads or reads with an unmapped pair; and green for reads that are overlaid and with the same start and end alignment positions. **C**. Various regions of or1384 were cloned under the T5 promoter in the vector pQE80oriT and expressed *in trans* in the WT to determine an effect on pigment levels. pNB46 contains the ORF preceded by the 5′UTR (−154 bp to +951 bp relative to the translation start codon); pNB47 contains the region where the peak of reads mapped in high abundance (−154 bp to +260 bp); and pNB48 contains only the ORF (+1 to +951).

While observing the transcriptional profile of the *pigA-O* cluster it was noted that a peak of reads mapped at the end of the cluster in the region around the translation start of a gene encoding a conserved hypothetical protein, transcribed divergently to *pigO*. A closer examination of this ORF (or1384; 951 bp; 316 aa) showed that the reads mapped predominantly to a region from −60 to +250 bp relative to the translation start in the replicates of WT and both the *hfq* and *rsmA* mutants (Figure 5A-B). The transcriptional profile suggested that the peak of reads could indicate the presence of a candidate *cis*-regulatory RNA element encoded within the mRNA of the CDS that regulates transcription elongation. The high abundance of reads in the UTR and 5′ region of or1384 followed by a significant and distinct drop in reads indicates that there is a high level of transcription initiation followed by a high rate of premature transcription termination about a quarter of the way into the CDS.

In order to investigate the function and regulation of the CDS, the region where reads mapped in high abundance (−154 bp to +260 bp), the ORF (+1 to +951) and the ORF preceded by the 5′ UTR (−154 bp to +951 bp) were cloned into plasmid vectors and expressed *in trans* in the WT to determine if there was an impact on prodigiosin production. Interestingly, pigment levels were increased when the CDS was expressed on its own whereas there was no effect when the CDS was expressed with the 5′ UTR or when the region spanning the peak of reads was expressed on its own (Figure 5C). Moreover, when overexpression of the vectors was induced by the addition of IPTG, only the vector expressing the CDS on its own caused a six-fold decrease in the maximal optical density of the culture whereas there was no effect for the vector with the ORF preceded by the 5′UTR. Therefore, the result may indicate the presence of a candidate novel *cis*-acting regulatory RNA element encoded within the 5′ UTR that prevents transcription elongation of the RNA transcript and expression of the protein.

The protein of or1384 is an uncharacterized protein predicted to contain two domains with the second C-terminal domain identified as conserved for the domain of unknown function PF04016 (DUF364) Pfam family (Additional file 1: Figure S2). Expression of the protein was confirmed by peptides mapping to or1384 detected by mass spectrometry in the iTRAQ proteomic experiment carried out as part of this study. The Dhaf4260 protein from *Desulfitobacterium hafniense* DCB-2 is the only solved protein structure of the DUF364 family, homologues of which are present in proteobacteria, firmicutes, actinobacteria, cyanobacteria, thermotogae and archaea [43]. The crystal structure of Dhaf4260 revealed that DUF364 is itself a novel combination of two well-known domains, an enolase N-terminal-like fold followed by a Rossmann-like domain, which form a unique catalytic site at the domain interface. An analysis of the genetic context and interdomain cleft suggests that DUF364 enzymes are involved in heavy metal transport and facilitate the synthesis of flavins, pterins, or similar compounds that chelate heavy metals [43]. Therefore it may be possible that the substrate of the or1384 product or another relevant metabolite is the cue detected by the candidate *cis*-regulatory element to regulate transcription elongation.

### Concluding remarks

In this study the transcriptome of S39006 WT and the *hfq* and *rsmA* mutants was characterized and differential expression determined. Transcriptome changes were correlated with altered protein expression that allowed for the characterization of the regulons of two major RNA-binding post-transcriptional regulators. Genes in the categories of secondary metabolism and motility were co-regulated and differentially expressed in both the *hfq* and *rsmA* mutants. Surprisingly, expression of the *pig* cluster was reduced at the level of RNA in both mutants but increased at the level of protein in the *rsmA* mutant, consistent with the hyperpigmented phenotype. This suggests that RsmA regulates translation of the Pig cluster. The second co-regulated category of genes includes the flagellar and chemotaxis genes which show elevated expression in both mutants. Increased flagellar gene expression is consistent with increased swimming and swarming motility in the *rsmA* mutant, whereas, paradoxically, the *hfq* mutant showed decreased motility. This indicates that *hfq* mutant cells are not able to coordinate movement using their flagella and suggests that Hfq plays a unique role in regulating flagellum production and movement in S39006 that is different from that seen in the taxonomically related *E. coli* and *S. typhimurium*. The differential expression analysis also identified Type II secretion and type IV pilus (T4P) systems and virulence factors that showed increased expression in the *rsmA* mutant and this was consistent with the increased virulence for this strain in both potato tuber rot and *C. elegans* infection assays. Surprisingly, RsmA regulated the activity of two mobile genetic elements, a pathogenicity island and a putative prophage, resulting in greater genomic flux in its absence. RsmA plays an important role in the regulation of virulence factors in several bacterial pathogens, but, to our knowledge, this is the first report that it also suppresses the activity of mobile genetic elements, assisting genome stability.

The candidate *cis*-acting regulatory RNA element explored in this study was shown to influence the expression of or1384. Expression of or1384 resulted in an increase of prodigiosin production, which was reduced when preceded by the UTR containing the putative *cis*-acting regulatory RNA element. Bioinformatic analysis of or1384 indicates that it most likely encodes an enzyme, and therefore one possibility is that the regulatory RNA element is a riboswitch that responds to a metabolic cue related to the function of the ORF. However, analysis of the UTR sequence revealed no matches to other known riboswitch secondary structures or sequences in Rfam [44].

In summary, this study characterized the regulons of Hfq and RsmA, controlled by two global regulatory RNA-binding proteins, at both an RNA and protein level using transcriptomic and proteomic methods, and has revealed new insights into their regulatory targets.

## Methods

### Bacterial strains and culture conditions

Bacterial strains used in this study include *Serratia* sp. ATCC 39006 *lacA* (WT) [6], and its derivatives NMW7 (*rsmA*::Tn) and NMW8 (*Δhfq*) [12]. S39006 WT and derivative strains were grown at 30°C in LB (5 g l^-1^ yeast extract, 10 g l^-1^ bacto tryptone and 5 g l^-1^ NaCl) at 200 r.p.m., or on LB agar supplemented with 1.5% (w/v) agar (LBA) [45]. Bacterial growth (OD_600_) was measured in a Unicam Helios spectrophotometer at 600 nm.

To test for infection by potential temperate prophage, 10 μl of filter-sterilized supernatant from cultures of WT and *rsmA*::Tn at early stationary phase growth (10 h, OD_600_ = 2.0 for WT) was mixed with 100 μl of culture of test strains and 4 ml 0.35% w/v bacto agar, and then poured onto LBA plates to create an overlay. Plates were incubated overnight at the appropriate temperature and examined for plaque formation. Strains tested included S39006, *S. marcescens* 274, *P. atrosepticum* SCRI1043, *P. carotovorum* subsp. *carotovorum*, *Citrobacter rodentium* DBS100, and *P. aeruginosa* PA01.

### DNA purification

All DNA manipulations were performed as previously described [46]. DNA was purified using DNA purification kits (Anachem) following the manufacturers’ instructions. Oligonucleotide primers used in this study are listed in Additional file 1: Tables S1 and S2. DNA sequencing was performed at the DNA Sequencing Facility, Department of Biochemistry, University of Cambridge. Nucleotide sequence data were analysed using BLAST and ClustalW [47, 48].

Genomic DNA was extracted using the DNAeasy Tissue kit (Qiagen). Bacteriophage DNA was extracted from filter-sterilized supernatant (450 μl) taken from cultures of WT and *rsmA*::Tn at early stationary phase growth. The Phase Lock Gel kit (Eppendorf) using 1.5 ml light phase tubes was used to extract phage DNA according to the manufacturer’s instructions.

### Total RNA extraction

RNA was extracted from cell cultures (2 ml) taken at early stationary phase (T = 10 hr, or OD_600_ = 2.0 for WT) growth in LB at 30°C and mixed with 4 mL RNA Later (Ambion). Total RNA was extracted using the hot phenol method as previously described [49]. Precipitated RNA was resuspended in 100 μl H_2_O and subjected to TURBO DNase digestion according to the manufacturer’s instructions (Ambion). The reaction was incubated at 37°C for 30 min. RNA was extracted with an equal volume of chloroform, precipitated with 2.5 volumes of ethanol, and dissolved in 50 μl H_2_O. PCR amplification of 16S rRNA before and after DNase digestion confirmed the removal of genomic DNA to below PCR-detectable levels. RNA was quantified using a Nanodrop ND1000 (NanoDrop Technologies) and the quality evaluated using an Agilent Bioanalyser (Agilent Technologies).

### qPCR

cDNA for quantitative RT-PCR was reverse transcribed from extracted RNA with random hexamers and qRT-PCR performed as described previously [17]. Minus reverse transcriptase (RT) controls were performed as a control for DNA contamination. The PCR amplification was performed with an ABI PRISM 7300 real-time PCR system and SYBR green PCR mix. Relative gene expression was obtained using 16S rRNA as the control with mRNA/16S rRNA = 1 in the WT. Oligonucleotides used as primers for quantitative PCR are listed in Additional file 1: Table S2.

qPCR on genomic DNA was performed as follows. 10 ng of genomic DNA was used as template in qPCR reactions. The PCR amplification was performed with an ABI PRISM 7300 real-time PCR system and SYBR green PCR mix. Relative copy number was determined using the delta-delta Ct method with *gyrB* as the reference gene. The ratio of copies of the sample to the reference gene was calculated as 2^Ct,sample^/2^Ct,gyrB^.

### Library preparation and Illumina sequencing

Single-stranded (ss) cDNA libraries were prepared and sequenced on the Illumina platform as described previously, but without depletion of 23S and 16S rRNA [50]. Briefly, 1 μg of RNA was used as template for reverse transcription with random hexamer primers and Superscript III reverse transcriptase (RT; Invitrogen) at 45°C for two hours. +RT and –RT reactions were assembled as controls. The + RT reactions were purified using an Illustra AutoSeq G50 spin column (GE Healthcare). PCR amplification of the transcribed genes of 16S rRNA (3916SF/R) and *pigB* (oNMW27-F/R) from the + RT but not –RT samples confirmed removal of genomic DNA from the RNA sample and successful generation of cDNAs. The synthesized cDNAs were assembled into paired-end sequencing libraries and sequenced on the Illumina GAIIX sequencer with a read length of 76 bp. The sequencing reads have been deposited in the Short Read Archive under the accession numbers ERS003527, 28, 29 (for WT replicates 1, 2, 3); ERS003530, 31, 32 (for *hfq* replicates 1, 2, 3); and ERS003533, 34, 35 (for *rsmA* replicates 1, 2, 3).

### Mapping of reads and differential expression analysis

Reads were mapped to the reference S39006 genome using the MAQ alignment program. Sequencing and assembly of the S39006 genome (~5 Mb) was carried out during the course of this study (Fineran et al., in press), and a draft version of the genome (accession no. AWXH01000000) was used for mapping of RNA-seq reads and protein identification. The DESeq package in the R software environment was used to calculate digital gene expression from the RNA-seq data and analyze differential gene expression between the WT and the mutants of *hfq* and *rsmA* using replicates 1 and 2 of WT, 1 and 3 of *hfq*, and 2 and 3 of *rsmA*. Of the three *rsmA* replicates, much fewer reads mapped from the first one and therefore two replicates per samples were used for consistency in the analysis. The DESeq package models count data using a negative binomial distribution [51]. *P* values were adjusted for multiple testing using the Benjamini-Hochberg false discovery rate (FDR) correction.

### Protein purification

In order to correlate transcriptomic and proteomic changes in the *hfq* mutant, triplicate cultures of WT and *hfq* were grown under the same conditions as samples subjected to RNA-seq. Cells from 25 ml cultures at early stationary phase growth in LB were pelleted by centrifugation. Cells were washed with 1 ml phosphate-buffered saline and the cell pellet resuspended in 1.01 ml CHAPS lysis buffer (8 M urea, 4% CHAPS (3-[(3-cholamidopropyl)dimethylammonio]-1-propane-sulphonate), 5 mM magnesium acetate, 10 mM Tris–HCl, pH 8.0) containing 1× protease inhibitor (protease inhibitor cocktail set I, Calbiochem). Cells were sonicated in 10 second bursts (10 seconds on and 20 seconds off) for 5 cycles. Samples were centrifuged first to pellet cell debris and insoluble material and a second time to ensure removal of insoluble material.

### Peptide labelling with iTRAQ

Protein samples were precipitated and labelled with isobaric tags for relative or absolute quantitation (iTRAQ) as previously described [52]. Chilled acetone (1.5 ml) was added to 250 μl of soluble protein sample and left at −20°C overnight. Precipitated proteins were collected by centrifugation (3,000 rpm, 10 min) and resuspended in iTRAQ labeling buffer (8 M urea, 2% Triton X100, 0.1% SDS and 25 mM triethyl ammonium bicarbonate (TEAB) pH 8.5). The detergent-compatible BCA protein assay (Pierce, Rockford, IL) was used to determine protein concentration.

iTRAQ labelling was performed using 100 μg protein from each sample or a pooled sample comprising equal amounts of each sample. The pooled protein sample served as an internal reference allowing for a comparison of relative quantitation. First, samples were reduced by the addition of TCEP (4 mM Tris(2-carboxyethyl) phosphine, 20°C, 1 h) and cysteines blocked by the addition of MMTS (8 mM methyl methanethiosulfonate, 20°C 10 minutes). Samples were diluted with 50 mM TEAB (pH 8.5) such that the final urea concentration was below 1 M, digested with trypsin overnight at 37°C (Promega; 2.5 μg added at 0 and 1 h) and lyophilized using a speed vacuum system.

Each lyophilized sample was resuspended in 100 μl labeling buffer (0.25 M TEAB, 75% ethanol), added to one unit of the corresponding iTRAQ reagent and incubated for 1 h at 20°C. Residual reagent was quenched by adding 100 μl water and incubated for a further 15 minutes at 20°C. The samples belonging to the iTRAQ comparisons were then pooled and lyophilized. Pool 1 consisted of samples WT-1, hfq-1, WT-2, and internal pooled sample labelled with iTRAQ tags 114, 115, 116, and 117, respectively. Pool 2 consisted of samples hfq-2, internal pooled sample, hfq-3, and WT-3 labelled with iTRAQ tags 114, 115, 116, and 117, respectively. Sample and pooled replicates were labelled with different tags to take into account the slight differences between the performance of the iTRAQ reporter ions in the mass spectrometer.

### Reverse-phase chromatography

Peptides were pre-fractionated by reverse-phase chromatography at high pH. The lyophilized samples were resuspended in 50 μl ammonium formate (20 mM, pH 10.0). The sample was loaded onto a C18 UPLC column (bridged ethyl hybrid, Waters, 2.1 × 150 mm column dimension, 1.7 μm particle size, 130 Å pore size). The peptides were separated and eluted using a 50 min linear gradient of 0-70% ACN at a flow rate of 0.25 ml/min. Eluted peptides were monitored with a diode array detector with a wavelength range of 200–400 nm and were manually collected in fractions at 2 min intervals. 30 fractions were collected per sample and reduced to dryness using a speed vacuum centrifuge. Fractions 8–26 for sample A and fractions 8–24 for sample B were analyzed by liquid chromatography followed by tandem mass spectrometry (LC-MS/MS). Fractions 25–26 for sample B were omitted because of very low peptide abundance.

### LC-MS/MS analysis

Peptide data collection was performed using a Q-Tof Premier tandem mass spectrometer (Waters) as described previously [53]. Samples were resuspended in 30 μl 0.1% formic acid. Peptides were separated by a second dimension of reverse-phase chromatography at low pH using a nanoAcquity HPLC pump (Waters) and a Waters Atlantis C18 analytical column (nanoAcquity) (Symmetry C18, 3 μM particle size, 75 μM i.d. × 100 mm). Peptides (1 μl) were injected using an auto sampler into a 10 μl sample loop and then loaded onto a pre-column (Waters Symmetry C18, 5 μM particle size, 180 μM i.d × 20 mm) with 0.1% formic acid at a flow rate of 5 μL/min for 4 minutes. Peptides were concentrated onto the pre-column and residual wash was directed to waste. After this period, the six port valve was switched to allow elution of the peptides at a flow rate of 300 nl/min from the pre-column onto the analytical column. The gradient employed was a 100 min linear gradient of 2-55% ACN. In MS mode, data were collected from *m/z* 400–1600. The instrument was run in data-dependent acquisition mode which means that following each MS scan, generally, the three most intense peptide ions were selected for MS/MS analysis. MS/MS data were collected between *m/z* 50–2000. Briefly, peptides were selected for MS/MS based on charge-state recognition (2+ and 3+). MS/MS data were collected for a total of 3 seconds, before the instrument was switched back to MS mode. Peptides which were selected for MS/MS analysis were excluded from re-selection for MS/MS for 300 s.

### MS data analysis and protein quantification

The QTof Premier raw files were converted into three file formats: 1) centroided pkl (peak list) format using ProteinLynx Global Server (PLGS) V2.4, 2) mzML format in order to obtain charge state information, and 3) mzXML format which is a universal MS file format created using Masswolf. The information contained in these three file formats was compiled to create an MGF (Mascot Generic Format) file. The MGF file was searched against the S39006 proteome database using the Mascot search algorithm to yield matches of MS/MS spectra to peptides. The S39006 proteome database was generated from the predicted ORFs in the reference genome. Within Mascot, the modifications used were as follows: fixed, iTRAQ 4-plex (K), iTRAQ 4-plex (N-term), methylthio modification (C); variable, oxidation (M), iTRAQ (Y). The MS tolerance was 1 Da, and the MS/MS tolerance was 0.8 Da. Each MGF file was also searched against a decoy proteome database to estimate the false discovery rate.

The search results outputted by Mascot were re-analyzed by Mascot Percolator, a machine learning algorithm that yields peptide/spectrum matches with a more robust false discovery rate [54]. The output of Mascot percolator and the corresponding mzXML file were inputted into iSPY [55], which matches peptide identifications from the MS/MS spectra with reporter ion peak areas to determine the relative abundance of the peptides. Results were filtered to apply a false discovery rate of 1%.

### Preparation and analysis of flagellar protein extracts

Flagellar protein extracts were prepared from S39006 grown to early stationary phase and analyzed by 1D SDS-PAGE. An aliquot of 25 ml cultures was taken and normalized to an OD = 2.0 in 5 ml. Cells were pelleted by centrifugation for 15 minutes at 4000 rpm (4°C) and resuspended in 5 ml PBS pH 7.4. The flagella were sheared from cells kept on ice using a T8 disperser/homogenizer (setting 3, 1 min). The cells were pelleted by centrifugation and protein in the supernatant was precipitated by the addition of 0.55 ml 100% trichloroacetic acid solution to a final concentration of 10% (w/v). Precipitated protein was pelleted by centrifugation at (17,000 rcf, 30 min, 4°C) and the pellet washed with ice cold acetone (1 ml). Protein pellets were resuspended in SDS-PAGE loading buffer (50 μl) and 20 μl analyzed by 10% 1D SDS-PAGE. Protein bands were identified by MALDI-TOF analysis (Protein & Nucleic Acid Chemistry Facility, Dept of Biochemistry).

### Electron microscopy

To prepare cells for examining the flagella by transmission electron microscopy (TEM), strains were grown to early stationary phase and an aliquot of culture was diluted to an OD 0.5 in 1 ml. Cells were pelleted gently by centrifugation at 3,000 rpm in a tabletop microcentrifuge to prevent shearing of flagella and were resuspended in 1 ml PBS.

To prepare phage for imaging, 50 ml cultures of S39006 WT and the NMW7 strain were grown to early stationary phase. Cells were pelleted by centrifugation and the supernatant filter sterilized using 0.22 uM filters. 45 ml of supernatant was applied to ultracentrifugation (16,500 rcf, 1 hr, 4°C). A small pellet was observed and the ultracentrifugation step was repeated (46,000 rcf, 1 hr, 4°C). The supernatant was decanted and the pellet washed with 15 ml of Lambda-dil (10 mM Tris pH 7.4, 5 mM MgSO_4_). Samples were applied to ultracentrifugation (48,900 rcf, 1 hr, 4°C). The supernatant was decanted and the pellet resuspended in 50 μl Lambda-dil.

EM grids were prepared with Formvar/carbon support films. 5 μl of slightly turbid suspension of cells or phage were pipetted directly onto the film side of the grid and allowed to settle for 30 seconds. An equal volume of the heavy metal stain 1% ammonium molybdate (+ 0.5% trehalose) was added to the grid and immediately drained with cut Whatman no. 1 filter paper. After air drying the grid was observed in the FEI Spirit Biotwin 120 kV transmission electron microscope and images captured on a F415 Tietz CCD camera.

### Plasmid construction and prodigiosin assay

To examine the influence of the UTR of or1384 on its expression, various regions of or1384 were amplified by PCR from genomic DNA and cloned into the *Eco*RI and *Hin*dIII sites under the IPTG-inducible T5 promoter (which has leaky expression) in the vector pQE80oriT. pNB46 contains the ORF preceded by the 5′UTR (−154 bp to +951 bp relative to the translation start codon); pNB47 contains the 5′UTR and some of the initial ORF (−154 bp to +260 bp); and pNB48 contains only the ORF (+1 to +951). Primers used for PCR are listed in Additional file 1: Table S1.

Plasmids were used to transform into *E. coli* β2163 and then transferred by conjugation into S39006 [56]. The assay for prodigiosin was performed as described previously [3].

## Electronic supplementary material

Additional file 1: Table S1: Oligonucleotide primers used in this study. **Table S2.** Oligonucleotide primers used for qPCR. **Table S3.** Comparison of the fold change (log_2_ ratio) between RNA-seq and qRT-PCR. **Table S4.** A selection of differentially expressed genes in the *hfq* and *rsmA* mutants. **Figure S1.** qRT-PCR of genes of interest. **Figure S2.** Secondary structure prediction of or1384. (DOCX 661 KB)

Additional file 2: Table S5: A complete list of all genes identified as differentially expressed in the RNA-seq or iTRAQ datasets for the hfq and rsmA mutants. (XLSX 248 KB)
