# Supplementary material for: RNA-seq reveals the RNA binding proteins, Hfq and RsmA, play various roles in virulence, antibiotic production and genomic flux in Serratia sp. ATCC 39006
Source: BMC Genomics. 2013 Nov 22;14(1):822. doi: 10.1186/1471-2164-14-822 (PMC4046660; doi:10.1186/1471-2164-14-822)
Supplement: Supplementary file 1 — Additional file 1: Table S1: Oligonucleotide primers used in this study. Table S2. Oligonucleotide primers used for qPCR. Table S3. Comparison of the fold change (log2 ratio) between RNA-seq and qRT-PCR. Table S4. A selection of differentially expressed genes in the hfq and rsmA mutants. Figure S1. qRT-PCR of genes of interest. Figure S2. Secondary structure prediction of or1384. (DOCX 661 KB) [file 12864_2013_5562_MOESM1_ESM.docx]

**Supplementary information**

Wilf *et al*., “RNA-seq reveals the RNA binding proteins, Hfq and RsmA, play various roles in virulence, antibiotic production and genomic flux in *Serratia* sp. 39006”

Table S1. Oligonucleotide primers used in this study. 1

Table S2. Oligonucleotide primers used for qPCR. 2

Table S3. Comparison of the fold change (log_2_ ratio) between RNA-seq and qRT-PCR. 4

Table S4. A selection of differentially expressed genes in the *hfq* and *rsmA* mutants. 5

Figure S1. qRT-PCR of genes of interest 14

Figure S2. Secondary structure prediction of or1384. 15

Table S1. Oligonucleotide primers used in this study.

| Name | Sequence (5’-3’) | Restriction Site | Notes |
| --- | --- | --- | --- |
| oNMW91-F | ATG^AATTCATTAAAGAGGAGAAATTAACTACAGATGAGCTCGAATAGTCG | *Eco*RI | F primer for cloning S39006 or1384 UTR + ORF into pQE80oriT (1105 bp, pNB46); rbs in blue |
| oNMW91-R | GATA^AGCTTTCAGCGCCCACAAAAG | *Hin*dIII | R primer for cloning S39006 or1384 UTR + ORF into pQE80oriT (1105 bp, pNB46) |
| oNMW92 | GATA^AGCTTCTTTTTGAGAAGCGCATACA | *Hin*dIII | R primer for cloning S39006 or1384 UTR into pQE80oriT (415 bp, pNB47) with oNMW91-F as F primer |
| oNMW93 | GATG^AATTCATTAAAGAGGAGAAATTAACTATGTGGTCTTCTGACGCAAC | *Eco*RI | F primer for cloning S39006 or1384 ORF (885 bp, pNB48) with oNMW91-R as R primer; rbs in blue |
| oNMW108-F | CCGGTGAATTGATTGTGTTCA |  | LHS primer out of prophage 3; overlap or4140 |
| oNMW108-R | ACAAGAGGAGGAATTGAAAGC |  | RHS primer out of prophage 3; within or4196 |
| oNMW109-F | CGTGCATTAAGCTTCATTGG |  | LHS primer into prophage 3 from genome; overlap or4139 |
| oNMW109-R | TCCGCGCTTAAACCAGAT |  | RHS primer into prophage 3 from genome; overlap or4197 |

Table S2. Oligonucleotide primers used for qPCR.

| Gene | Sequence (5’-3’) | Name |
| --- | --- | --- |
| *16S rRNA* | GGCCTTCGGGTTGTAAAGTC | 3916SF |
| *16S rRNA* | GCTTTACGCCCAGTCATTC | 3916SR |
| *gyrB* | CGTCTGCGTGAGTTGTCATTC | oNMW28-F |
| *gyrB* | CGGTGTCTTGTTACGGTTGAG | oNMW28-R |
| *pigA* | GGATGCCATTCAAACTTTTGG | oNMW76-F |
| *pigA* | TTGGTACCGGAGAAAATTCGA | oNMW76-R |
| *pigB* | TGATGATGGCTGTCTTTGTG | oNMW27-F |
| *pigB* | GTGGTCGGGAAGGTTCTC | oNMW27-R |
| *carA* | GGAGAATTCTGCTTCTTGATTG | qcarAf |
| *carA* | CTGACAAACCCGATCTTCAC | qcarAr |
| *carR* | CCATTTTCCTGGGATGATAAC | qcarRf |
| *carR* | TAGTCAGGATCGCCAGATTG | qcarRr |
| *smaI* | TGCCTGTGGATGAGCATAAC | oNMW75-F |
| *smaI* | TCCCTGACGCAGAATGATAG | oNMW75-R |
| *smaR* | GACAAATCCTGCGATGATGA | oNMW65-F |
| *smaR* | CCATGCTTGCCCAGTAAAGT | oNMW65-R |
| *luxS* | TGGCGTGGAAGTTGTTGATA | oNMW66-F |
| *luxS* | CATTGAGCTCCGGGATTTTA | oNMW66-R |
| *rpoS* | AGGATTCCGCTTTTCAACCT | oNMW61-F |
| *rpoS* | CTATCGAGCTGTTCGGCAAT | oNMW61-R |
| *rsmA* | GCGAAACCCTCATGATTG | qrsmAf |
| *rsmA* | CAATAAGATGTTGGCTGAGACTTC | qrsmAr |
| *rhlA* | GGTGAGTCACTGGAACATAAT | RHLAF |
| *rhlA* | ACAAACCACCCCATGAAAT | RHLAR |
| *flhC* | CGAAAGTGAAACGCAATTAAG | FLHCF |
| *flhC* | TACCGCCTCAACGCTACTAC | FLHCR |
| *or3030* | AAATTGTACGGGCAGGACAG | oNMW104-F |
| *or3030* | TGGAAACTGAGCGTGAACTG | oNMW104-R |
| *or2773* | ATTAACGCCCTCGAACACAC | oNMW105-F |
| *or2772* | CAGGCTTTTTGCAGCATGTA | oNMW105-R |
| *or1483* | GCTGCACTGGCTGAATACAA | oNMW106-F |
| *or1483* | GGTAAACCAGCATTGGCTCA | oNMW106-R |
| *or4140* | ACCGGATGATGTGACACTGA | oNMW107-F |
| *or4140* | TTCCTTGTCTGTTGCCAATG | oNMW107-R |
| Prophage 3 *attP* | GTGGTGTGAATGCGAGTAATC | oNMW110 |
| Prophage 3 *attP* | ACAAGAGGAGGAATTGAAAGC | oNMW108-R |
| PAI *attP* | ATCTCAGGATGCGAACTTGC | oNMW111-F |
| PAI *attP* | TCATGCCCGTTAAGGATCTC | oNMW111-R |
| *or3032* | CAGCACTATCTGCCCAGTCA | oNMW115-F |
| *or3032* | CAGAGGCAATCAGCAGTGAA | oNMW115-R |
| *or3031* | GAACCGATTAGCCCGATACA | oNMW116-F |
| *or3031* | CAGATCGACAGGTACGCAGA | oNMW116-R |

Table S3. Comparison of the fold change (log_2_ ratio) between RNA-seq and qRT-PCR.

|  | *hfq*/WT |  |  |  | *rsmA*/WT |  |  |  |
| --- | --- | --- | --- | --- | --- | --- | --- | --- |
| Gene | qPCR fold change | *P* val | RNA-seq fold change | adj  *P* val^1^ | qPCR fold change | *P* val | RNA-seq fold change | adj  *P* val^1^ |
| *gyrB* | 0.18 | <0.5 | 0.18 | <1 | -0.32 | <0.35 | 0.12 | <0.8 |
| *pigA* | -6.17 | <0.05 | -3.13 | <0.001 | -0.56 | <0.35 | -0.98 | <0.001 |
| *pigB* | -5.91 | <0.01 | -2.22 | <0.01 | -0.99 | <0.05 | -1.21 | <0.001 |
| *carA* | -8.76 | <0.01 | -5.02 | <0.001 | ⎯ | ⎯ | ⎯ | ⎯ |
| *smaI* | 0.01 | <1 | 0.18 | <1 | -0.38 | <0.5 | 0.19 | <0.9 |
| *smaR* | -1.42 | <0.05 | 0.22 | <1 | -1.36 | <0.05 | -0.59 | <0.6 |
| *carR* | -5.14 | <0.0001 | -4.10 | <0.05 | ⎯ | ⎯ | ⎯ | ⎯ |
| *luxS* | 0.22 | <0.7 | -0.06 | <1 | ⎯ | ⎯ | ⎯ | ⎯ |
| *rpoS* | -1.60 | <0.05 | -1.50 | <0.01 | ⎯ | ⎯ | ⎯ | ⎯ |
| *ompA* | -0.26 | <0.5 | 0.74 | <0.85 | ⎯ | ⎯ | ⎯ | ⎯ |
| *ompX* | 0.06 | <0.9 | 0.55 | <0.7 | ⎯ | ⎯ | ⎯ | ⎯ |
| *rsmA* | 0.27 | <0.45 | 0.29 | <1 | ⎯ | ⎯ | ⎯ | ⎯ |
| *rhlA* | -2.90 | <0.05 | -0.82 | <0.9 | 5.23 | <0.0001 | 2.23 | <0.001 |
| *flhC* | 1.37 | <0.1 | 1.14 | <0.35 | 2.29 | <0.0001 | 2.24 | <0.001 |

^1^ *P* values were adjusted for multiple testing according to the method of Benjamini-Hochberg.

Table S4. A selection of differentially expressed genes in the *hfq* and *rsmA* mutants.

| Gene | *hfq* RNA^1^ | *hfq* Protein^1^ | *rsmA* RNA^1^ | *rsmA* Protein^1^ | Description |
| --- | --- | --- | --- | --- | --- |
| Pig cluster | | | | | |
| or1369 | 0.11 | 0.24 | 0.51 | 1.71 | PigA; |
| or1370 | 0.21 | ⎯ | 0.43 | 1.96 | PigB; |
| or1371 | 0.26 | 0.37 | 0.57 | ⎯ | PigC; |
| or1372 | 0.27 | 0.32 | 0.68 | ⎯ | PigD; |
| or1373 | ⎯ | 0.32 | ⎯ | ⎯ | PigE; |
| or1374 | ⎯ | 0.41 | ⎯ | 1.55 | PigF; |
| or1376 | ⎯ | ⎯ | 0.67 | ⎯ | PigH; |
| or1384 | ⎯ | ⎯ | 0.56 | ⎯ | Uncharacterized protein after the end of the *pig* cluster; |
| Car cluster | | | | | |
| or3484 | 0.06 | ⎯ | ⎯ | ⎯ | CarR; |
| or3483 | 0.03 | 0.18 | 0.30 | 0.51 | CarA; |
| or3482 | 0.05 | 0.17 | 0.31 | 0.46 | CarB; |
| or3481 | 0.01 | 0.18 | 0.21 | 0.47 | CarC; |
| or3480 | 0.16 | ⎯ | 0.40 | ⎯ | CarD; |
| or3478 | 0.13 | ⎯ | 0.22 | ⎯ | CarF; |
| Additional secondary metabolism genes | | | | | |
| or1961 | ⎯ | ⎯ | 2.31 | ⎯ | Non-ribosomal peptide synthase; |
| or1962 | ⎯ | ⎯ | 2.63 | ⎯ | Polyketide synthase; |
| or1963 | ⎯ | ⎯ | 3.21 | ⎯ | Non-ribosomal peptide synthase; |
| or1937 | ⎯ | ⎯ | 0.36 | ⎯ | Putative non-ribosomal peptide synthesis thioesterase; |
| or2773 | ⎯ | ⎯ | 2.30 | ⎯ | Similar to proteins involved in antibiotic biosynthesis; |
| Regulators of prodigiosin production | | | | | |
| or0460 | ⎯ | ⎯ | 0.53 | ⎯ | PhoU protein; |
| or0464 | ⎯ | ⎯ | 0.65 | 0.48 | PstS protein; |
| or3380 | 3.39 | 2.85 | ⎯ | ⎯ | PigU, Transcriptional regulator, LysR family; |
| or3017 | ⎯ | ⎯ | 0.34 | 0.68 | Rap, Transcriptional regulator slyA; |
| or0693 | ⎯ | ⎯ | 0.49 | ⎯ | VfmE, Transcriptional regulator, AraC family; |
| Protein export | | | | | |
| or0052 | ⎯ | ⎯ | 0.59 | ⎯ | Preprotein translocase subunit secY; |
| or0772 | 2.49 | ⎯ | ⎯ | ⎯ | Preprotein translocase, SecE subunit; |
| Type II secretion system | | | | | |
| or3465 | ⎯ | ⎯ | 1.92 | ⎯ | OutD; |
| or3464 | ⎯ | ⎯ | 2.08 | ⎯ | OutE; |
| or3461 | ⎯ | ⎯ | 2.43 | ⎯ | OutH; |
| or3460 | ⎯ | ⎯ | 2.99 | ⎯ | OutI; |
| or3856 | ⎯ | ⎯ | 2.51 | ⎯ | Putative major pilin subunit; |
| or3857 | ⎯ | ⎯ | 3.15 | ⎯ | Type II secretion system protein E; |
| or3858 | ⎯ | ⎯ | 1.81 | ⎯ | Type II secretion system protein; |
| Type IV pilus system | | | | | |
| or0865 | ⎯ | ⎯ | 1.57 | ⎯ | Type IV pilus secretin PilQ; |
| or1481 | ⎯ | ⎯ | 1.63 | ⎯ | PilM, type IV pilus gene cluster |
| or1483 | ⎯ | ⎯ | 1.65 | ⎯ | PilO, type IV pilus gene cluster |
| or1484 | ⎯ | ⎯ | 1.47 | ⎯ | PilP, type IV pilus gene cluster |
| or1485 | ⎯ | ⎯ | 1.44 | ⎯ | PilQ, type IV pilus gene cluster |
| or1490 | ⎯ | ⎯ | 1.52 | ⎯ | PilV, bacterial shufflon protein, type IV pilus gene cluster |
| Virulence factors | | | | | |
| or0146 | ⎯ | ⎯ | 0.49 | ⎯ | Pectate lyase/Amb allergen; |
| or0147 | 0.37 | ⎯ | 0.43 | ⎯ | Pectate lyase/Amb allergen; |
| or2371 | ⎯ | ⎯ | 0.71 | ⎯ | Pectate lyase; |
| or2779 | ⎯ | ⎯ | 4.67 | ⎯ | Pectate lyase L; |
| or2780 | ⎯ | ⎯ | 0.36 | ⎯ | Cellulase Z; |
| or2784 | ⎯ | 2.25 | ⎯ | ⎯ | Putative virulence effector protein SrfB; |
| or2783 | 3.46 | ⎯ | ⎯ | ⎯ | Putative virulence effector protein SrfA; |
| or1354 | ⎯ | ⎯ | 183.69 | ⎯ | Putative thermostable hemolysin; |
| Sigma factors | | | | | |
| or0342 | ⎯ | ⎯ | 0.49 | ⎯ | RpoH, heat shock sigma factor; |
| or0880 | ⎯ | ⎯ | 0.66 | ⎯ | RpoN, nitrogen-limitation sigma factor; |
| or1080 | ⎯ | ⎯ | 0.44 | ⎯ | RpoD, housekeeping sigma factor; |
| or3150 | ⎯ | ⎯ | 3.13 | ⎯ | RpoF, flagellar sigma factor; |
| or4255 | 0.35 | ⎯ | 0.34 | 0.27 | RpoS, starvation/stationary phase sigma factor; |
| Stress response | | | | | |
| or0679 | ⎯ | ⎯ | 0.42 | ⎯ | Superoxide dismutase; |
| or2835 | 2.41 | ⎯ | 0.65 | ⎯ | Tellurite resistance protein; |
| or2834 | ⎯ | ⎯ | 0.61 | ⎯ | Tellurium resistance protein TerA; |
| or2832 | ⎯ | ⎯ | 0.49 | ⎯ | Integral membrane protein TerC; |
| or2831 | ⎯ | ⎯ | 0.54 | ⎯ | Tellurium resistance protein terD; |
| Outer membrane proteins | | | | | |
| or1613 | ⎯ | ⎯ | 0.49 | 0.57 | Outer membrane chaperone Skp (OmpH); |
| or2177 | ⎯ | ⎯ | 0.44 | ⎯ | Virulence-related outer membrane protein; |
| or3566 | ⎯ | ⎯ | 0.38 | ⎯ | OmpA domain protein transmembrane region-containing protein; |
| or3503 | ⎯ | ⎯ | 12.38 | ⎯ | Polysaccharide biosynthesis protein; |
| or3039 | ⎯ | ⎯ | 4.94 | ⎯ | Porin LamB type; |
| or3016 | ⎯ | ⎯ | 0.38 | ⎯ | Outer membrane lipoprotein Pcp; |
| or2896 | ⎯ | ⎯ | 0.66 | ⎯ | Filamentous hemagglutinin family outer membrane protein; |
| or2702 | ⎯ | ⎯ | 0.51 | ⎯ | Outer membrane protein W; |
| or3762 | 2.64 | ⎯ | ⎯ | ⎯ | Outer membrane assembly lipoprotein YfiO; |
| Efflux pumps | | | | | |
| or1355 | ⎯ | ⎯ | 7.52 | ⎯ | Drug resistance transporter, Bcr/CflA subfamily; |
| or1930 | ⎯ | ⎯ | 1.60 | ⎯ | Probable multidrug efflux system transmembrane protein; |
| or1938 | 0.16 | ⎯ | 0.11 | ⎯ | Putative Drug/metabolite exporter family; |
| or1960 | ⎯ | ⎯ | 2.24 | ⎯ | Inner membrane component of tripartite multidrug resistance system; |
| or3320 | ⎯ | ⎯ | 2.05 | ⎯ | Drug resistance transporter, Bcr/CflA subfamily; |
| or3318 | ⎯ | ⎯ | 1.69 | ⎯ | Efflux transporter, RND family, MFP subunit; |
| or3317 | ⎯ | ⎯ | 1.49 | ⎯ | Transporter, hydrophobe/amphiphile efflux-1 (HAE1) family; |
| or3316 | ⎯ | ⎯ | 1.69 | ⎯ | RND efflux system, outer membrane lipoprotein, NodT family; |
| or3788 | ⎯ | ⎯ | 0.53 | ⎯ | Outer membrane efflux protein; |
| Electron transport | | | | | |
| or3379 | 3.53 | ⎯ | ⎯ | ⎯ | NADH-quinone oxidoreductase subunit A; |
| or3378 | 3.23 | ⎯ | ⎯ | ⎯ | NADH-quinone oxidoreductase, B subunit; |
| or3377 | ⎯ | ⎯ | 0.56 | ⎯ | NADH-quinone oxidoreductase subunit C/D; |
| or3376 | 2.20 | ⎯ | ⎯ | ⎯ | NADH-quinone oxidoreductase, E subunit; |
| or3375 | 2.38 | ⎯ | ⎯ | ⎯ | NADH-quinone oxidoreductase, F subunit; |
| or3373 | 2.90 | ⎯ | 0.62 | ⎯ | NADH dehydrogenase I subunit H; |
| or3371 | ⎯ | ⎯ | 0.44 | ⎯ | NADH dehydrogenase (Quinone); |
| or3370 | 2.98 | ⎯ | ⎯ | ⎯ | NADH-quinone oxidoreductase subunit K; |
| or3368 | 3.05 | ⎯ | ⎯ | ⎯ | Proton-translocating NADH-quinone oxidoreductase, chain M; |
| or3367 | ⎯ | ⎯ | 0.30 | ⎯ | Proton-translocating NADH-quinone oxidoreductase, chain N; |
| Flagellar and chemotaxis motility locus | | | | | |
| or3205 | ⎯ | 1.63 | 0.36 | ⎯ | Methyl-accepting chemotaxis sensory transducer; |
| or3204 | 4.63 | ⎯ | 5.43 | ⎯ | Flagellar transcriptional activator; |
| or3203 | ⎯ | ⎯ | 4.73 | ⎯ | Flagellar transcriptional activator FlhC; |
| or3200 | 3.40 | ⎯ | ⎯ | 1.90 | CheA signal transduction histidine kinase; |
| or3199 | 4.10 | ⎯ | ⎯ | 2.05 | CheW protein; |
| or3198 | 3.83 | ⎯ | 2.35 | 2.20 | Methyl-accepting chemotaxis sensory transducer; |
| or3197 | 4.52 | ⎯ | ⎯ | 1.78 | MCP methyltransferase, CheR-type; |
| or3196 | 2.47 | ⎯ | ⎯ | ⎯ | Response regulator receiver modulated CheB methylesterase; |
| or3195 | 3.93 | ⎯ | ⎯ | ⎯ | Chemotaxis protein; |
| or3194 | 2.78 | ⎯ | 1.89 | ⎯ | Chemotaxis phosphatase, CheZ; |
| or3192 | 3.11 | ⎯ | 1.86 | ⎯ | FlhA protein; |
| or3191 | 4.27 | ⎯ | ⎯ | ⎯ | FlhE protein; |
| or3190 | 2.87 | ⎯ | ⎯ | ⎯ | FlgN family protein; |
| or3187 | 7.98 | ⎯ | 4.00 | ⎯ | Flagellar basal-body rod protein; |
| or3186 | 9.14 | ⎯ | 2.99 | ⎯ | Flagellar basal-body rod protein FlgC; |
| or3185 | 8.15 | ⎯ | 2.94 | ⎯ | Flagellar hook capping protein; |
| or3184 | 6.34 | ⎯ | 1.70 | ⎯ | Fagellar hook-basal body protein; |
| or3183 | 6.64 | ⎯ | 2.38 | ⎯ | Flagellar basal-body rod protein FlgF; |
| or3182 | 5.32 | ⎯ | 2.35 | ⎯ | Flagellar basal-body rod protein FlgG; |
| or3181 | 5.27 | ⎯ | 1.87 | ⎯ | Flagellar L-ring protein; |
| or3180 | 4.77 | ⎯ | 2.33 | ⎯ | Flagellar P-ring protein; |
| or3179 | 4.73 | ⎯ | 1.88 | ⎯ | Flagellar rod assembly protein/muramidase FlgJ; |
| or3178 | 2.55 | ⎯ | 1.62 | ⎯ | Flagellar hook-associated protein FlgK; |
| or3177 | 3.85 | ⎯ | 1.72 | ⎯ | Flagellar hook-associated protein 3; |
| or3171 | 3.40 | ⎯ | ⎯ | ⎯ | Flagellar motor switch protein FliM; |
| or3170 | 3.10 | ⎯ | ⎯ | ⎯ | Flagellar basal body-associated protein FliL; |
| or3169 | 4.69 | ⎯ | ⎯ | ⎯ | Flagellar hook-length control protein; |
| or3167 | 2.67 | ⎯ | 2.03 | ⎯ | Flagellar protein export ATPase FliI; |
| or3166 | 4.73 | ⎯ | 2.44 | ⎯ | Flagellar assembly protein FliH/Type III secretion system HrpE; |
| or3165 | ⎯ | ⎯ | 2.37 | ⎯ | Flagellar motor switch protein FliG; |
| or3164 | 5.64 | ⎯ | 2.21 | ⎯ | Flagellar M-ring protein FliF; |
| or3163 | 8.26 | ⎯ | 3.93 | ⎯ | Flagellar hook-basal body complex protein fliE; |
| or3161 | ⎯ | ⎯ | 2.73 | ⎯ | Flagellar protein; |
| or3160 | 3.65 | ⎯ | 1.62 | ⎯ | Flagellar hook-associated protein 2; |
| or3159 | ⎯ | ⎯ | ⎯ | 2.80 | Flagellin; |
| or3149 | 3.38 | ⎯ | 4.46 | ⎯ | Protein FliZ; |
| Putative prophage 1 | | | | | |
| or2488 | 2.45 | ⎯ | ⎯ | ⎯ | Tail E family protein; |
| or2490 | ⎯ | ⎯ | ⎯ | 1.88 | Tail sheath protein; |
| or2502 | 3.43 | ⎯ | ⎯ | ⎯ | Tail assembly chaperone gp38; |
| or2503 | 3.27 | ⎯ | ⎯ | ⎯ | Tail Collar domain protein; |
| or2520 | ⎯ | ⎯ | 2.21 | ⎯ | CI repressor; |
| or2552 | ⎯ | ⎯ | 0.46 | 0.57 | Integration host factor subunit alpha; |
| Putative prophage 2 | | | | | |
| or2650 | ⎯ | ⎯ | 1.87 | ⎯ | Putative uncharacterized protein; |
| or2648 | ⎯ | ⎯ | 1.65 | ⎯ | Putative uncharacterized protein; |
| or2636 | ⎯ | ⎯ | 1.58 | ⎯ | Putative uncharacterized protein; |
| or2635 | ⎯ | ⎯ | 1.68 | ⎯ | Putative uncharacterized protein; |
| or2633 | ⎯ | ⎯ | 1.71 | ⎯ | Putative uncharacterized protein; |
| or2628 | ⎯ | ⎯ | 1.56 | ⎯ | Putative uncharacterized protein; |
| or2626 | ⎯ | ⎯ | 1.47 | ⎯ | Phage-associated protein, HI1409 family; |
| or2623 | ⎯ | ⎯ | 1.79 | ⎯ | Putative uncharacterized protein; |
| or2615 | ⎯ | ⎯ | 1.88 | ⎯ | Lysozyme; |
| or3800 | ⎯ | ⎯ | 1.97 | 1.83 | Single-stranded DNA-binding protein; |
| or3801 | ⎯ | ⎯ | 7.68 | ⎯ | RNA-directed DNA polymerase; |
| Putative prophage 3 | | | | | |
| or4140 | ⎯ | ⎯ | 3.10 | ⎯ | Tail assembly chaperone gp38; |
| or4141 | ⎯ | ⎯ | 3.90 | ⎯ | Putative tail fiber protein; |
| or4142 | ⎯ | ⎯ | 2.86 | ⎯ | Putative bacteriophage protein; |
| or4143 | ⎯ | ⎯ | 2.65 | ⎯ | Putative bacteriophage protein; |
| or4144 | ⎯ | ⎯ | 2.13 | ⎯ | Putative bacteriophage protein; |
| or4145 | ⎯ | ⎯ | 2.45 | ⎯ | Putative bacteriophage protein; |
| or4149 | ⎯ | ⎯ | 2.06 | ⎯ | Putative uncharacterized protein; |
| or4150 | ⎯ | ⎯ | 1.94 | ⎯ | Putative bacteriophage protein; |
| or4151 | ⎯ | ⎯ | 2.20 | ⎯ | Putative uncharacterized protein; |
| or4152 | ⎯ | ⎯ | 2.25 | ⎯ | Lytic transglycosylase, catalytic; |
| or4153 | ⎯ | ⎯ | 2.44 | ⎯ | Putative uncharacterized protein; |
| or4154 | ⎯ | ⎯ | 2.61 | ⎯ | Putative bacteriophage protein; |
| or4155 | ⎯ | ⎯ | 2.01 | ⎯ | Putative uncharacterized protein; |
| or4156 | ⎯ | ⎯ | 2.07 | ⎯ | Putative bacteriophage protein; |
| or4157 | ⎯ | ⎯ | 2.10 | ⎯ | Putative uncharacterized protein; |
| or4158 | ⎯ | ⎯ | 1.98 | ⎯ | Putative bacteriophage protein; |
| or4159 | ⎯ | ⎯ | 1.93 | ⎯ | Hypothetical phage protein; |
| or4160 | ⎯ | ⎯ | 1.72 | ⎯ | Putative bacteriophage protein; |
| or4161 | ⎯ | ⎯ | 2.05 | ⎯ | Putative bacteriophage protein; |
| or4162 | ⎯ | ⎯ | 2.17 | ⎯ | Putative bacteriophage protein; |
| or4163 | ⎯ | ⎯ | 2.24 | ⎯ | Putative uncharacterized protein STY2037; |
| or4164 | ⎯ | ⎯ | 2.01 | ⎯ | Putative bacteriophage protein; |
| or4165 | ⎯ | ⎯ | 2.11 | ⎯ | Phage-associated protein, family; |
| or4166 | ⎯ | ⎯ | 1.69 | ⎯ | Putative bacteriophage protein; |
| or4167 | ⎯ | ⎯ | 1.72 | ⎯ | Putative bacteriophage protein; |
| or4169 | ⎯ | ⎯ | 2.30 | ⎯ | Predicted CDS Pa_2_6160; |
| or4170 | ⎯ | ⎯ | 1.83 | ⎯ | Putative uncharacterized protein; |
| or4171 | ⎯ | ⎯ | 1.69 | ⎯ | Lysozyme; |
| or4173 | ⎯ | ⎯ | 1.72 | ⎯ | Putative DNA adenine methylase; |
| or4174 | ⎯ | ⎯ | 1.70 | ⎯ | Gifsy-1 prophage RegQ; |
| or4177 | ⎯ | ⎯ | 1.67 | ⎯ | NinB protein; |
| or4179 | ⎯ | ⎯ | 1.57 | ⎯ | Putative cytosine-specific modification methylase; |
| or4183 | ⎯ | ⎯ | 1.63 | ⎯ | DNA replication protein DnaC; |
| or4184 | ⎯ | ⎯ | 1.65 | ⎯ | Conserved domain protein; |
| or4185 | ⎯ | ⎯ | 1.71 | ⎯ | Predicted protein; |
| or4186 | ⎯ | ⎯ | 1.69 | ⎯ | Regulatory protein CII; |
| or4192 | ⎯ | ⎯ | 1.70 | ⎯ | DNA polymerase III, epsilon subunit; |
| or4194 | ⎯ | ⎯ | 1.52 | ⎯ | Putative uncharacterized protein; |
| or4196 | ⎯ | ⎯ | 1.56 | ⎯ | Putative phage integrase; |
| Additional genes of interest | | | | | |
| or0363 | ⎯ | ⎯ | 1.49 | ⎯ | Cellulose synthase operon C domain protein; |
| or0697 | ⎯ | ⎯ | 0.11 | ⎯ | Gas vesicle protein GvpA1; |
| or1348 | ⎯ | 0.65 | 0.44 | ⎯ | QueF, 7-cyano-7-deazaguanine reductase, queuosine biosynthesis; |
| or1352 | ⎯ | ⎯ | 8.04 | ⎯ | Taurine catabolism dioxygenase TauD/TfdA; |
| or1353 | ⎯ | ⎯ | 12.62 | ⎯ | AMP-dependent synthetase and ligase; |
| or1713 | ⎯ | ⎯ | 83.17 | 21.22 | Putative exported protein; |
| or2279 | ⎯ | ⎯ | 4.68 | 9.67 | RhlA, surfactant biosynthesis; |
| or2684 | ⎯ | ⎯ | 0.37 | 0.68 | H-NS protein; |

^1^Fold changes were calculated as the ratio of mutant to WT. Differentially expressed genes were identified with an FDR threshold of 10% for RNA levels of *hfq* and protein levels of *rsmA*. A 5% FDR threshold was used for RNA levels of *rsmA*. *hfq* protein levels were identified with a *P* value < 10%.


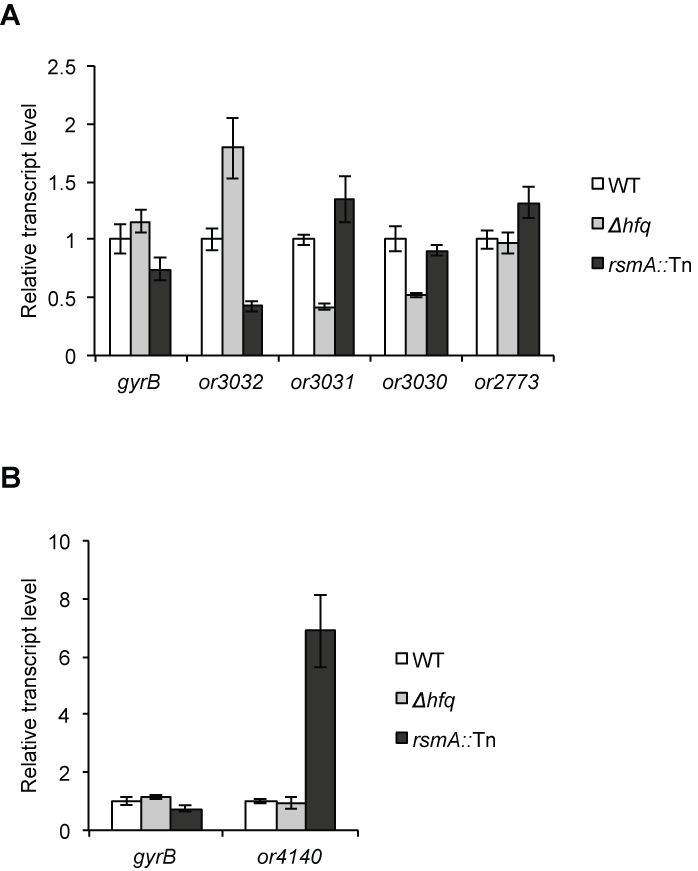


Figure S1. qRT-PCR of genes of interest

Gene expression for *Δhfq* (A) and *rsmA*::Tn (B) is measured as transcript levels relative to WT at early stationary phase growth. *gyrB* is included as a negative control. Values represent average gene expression ± SD from three independent experiments.


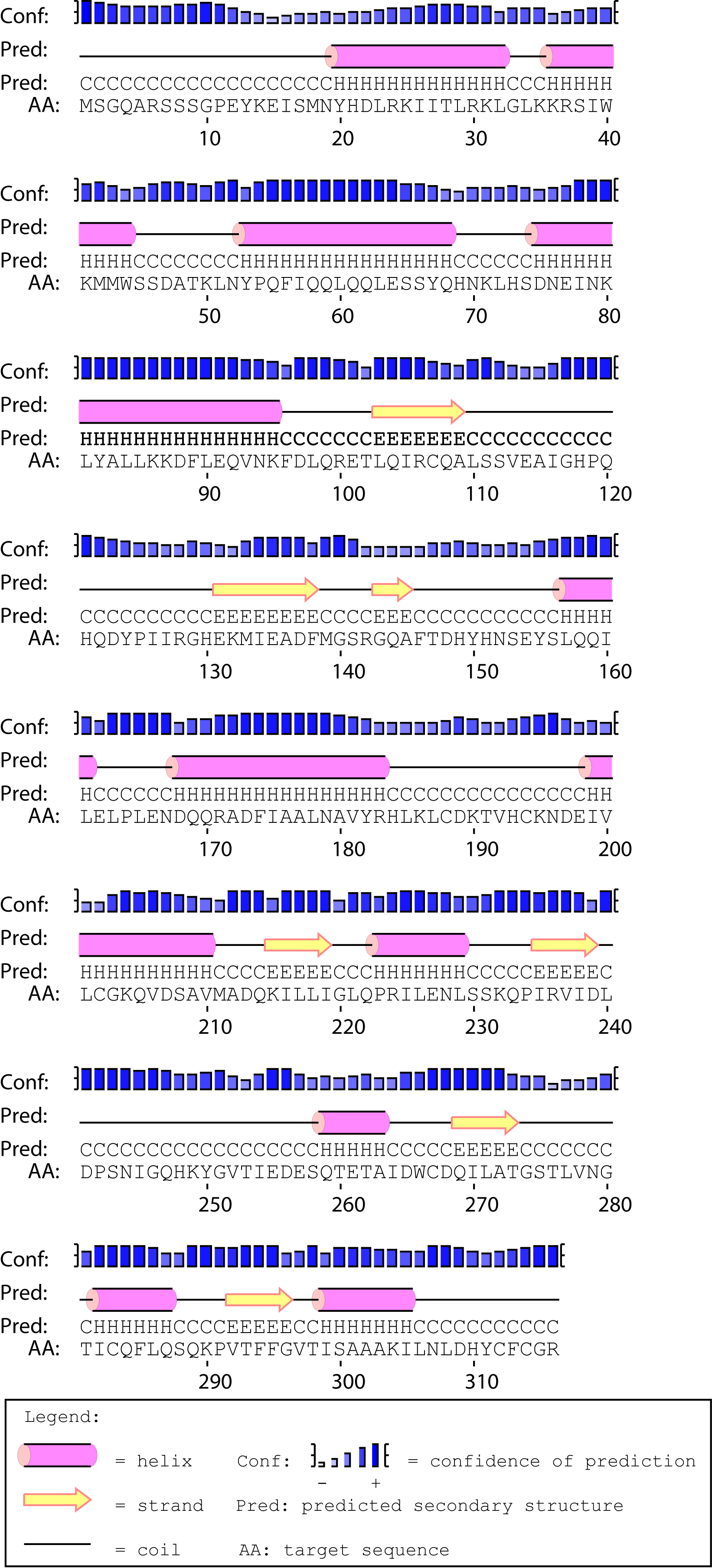


Figure S2. Secondary structure prediction of or1384.

The secondary structure of or1384 (316 aa) was predicted by PSIPRED and the graphical diagram created by DomPred. Two domains were predicted with a domain boundary at aa 88, and aa 140-309 was conserved for DUF364.
